# Fat-containing soft-tissue tumors: Imaging findings and pathologic correlation

**DOI:** 10.1007/s00256-026-05160-z

**Published:** 2026-02-20

**Authors:** Leonor G. Savarese, Nicolas Papalexis, Mateus A. Hernandes, Paolo Spinnato, Joel Del Bel Pádua, Giancarlo Facchini, Nelson F. Gava, Edgard E. Engel, Marcello Henrique Nogueira-Barbosa

**Affiliations:** 1 Department of Medical Imaging, Hematology and Clinical Oncology, Ribeirao Preto Medical School, University of Sao Paulo, Av. Bandeirantes, 3900 CEP, Ribeirão Preto, SP 14049-090 Brazil; 2 Department of Radiology, ARNAS G. Brotzu – Businco Oncologic Hospital, Cagliari, Italy; 3 Diagnostic and Interventional Radiology Unit, IRCCS Istituto Ortopedico Rizzoli, Bologna, Italy; 4 Department of Pathology and Forensic Medicine, Ribeirao Preto Medical School, University of Sao Paulo, Ribeirão Preto, São Paulo Brazil; 5 Department of Orthopedics and Anesthesiology, Ribeirao Preto Medical School, University of Sao Paulo, Ribeirão Preto, São Paulo Brazil

**Keywords:** Lipoma, Liposarcoma, Magnetic resonance imaging, Fat, Soft-tissue tumor, MRI, Neoplasm

## Abstract

Fat-containing soft-tissue tumors encompass a broad spectrum of entities, ranging from indolent lipomas to aggressive liposarcomas, many of which share overlapping MRI features that pose diagnostic challenges even for experienced radiologists. In this review, we provide a focused, evidence-based synthesis of the current literature to outline a practical framework for the imaging evaluation of adipocytic soft-tissue lesions. Key MRI features that aid in distinguishing benign from intermediate and malignant tumors are discussed, with emphasis on imaging–pathology correlation and common diagnostic pitfalls. While conventional MRI criteria, such as lesion size, depth, septal thickness, and nodularity, remain central to risk stratification, we also review the complementary role of contrast-enhanced MRI and molecular testing, including MDM2 amplification and FUS::DDIT3 fusion analysis, particularly in indeterminate cases. Emerging tools, such as radiomics and artificial intelligence–based approaches, are briefly addressed as evolving adjuncts. By integrating classical imaging principles with contemporary classification frameworks, this article aims to serve as a comprehensive and clinically relevant reference for radiologists involved in the assessment and management of fat-containing soft-tissue tumors.

## Introduction

Fat-containing soft-tissue tumors represent one of the most common groups of mesenchymal neoplasms, encompassing lesions that range from benign lipomas to high-grade liposarcomas, each with distinct biological behavior and prognostic implications [1–3].

Ultrasonography is generally the first-line modality for evaluating superficial adipocytic lesions [4]. MRI serves as the gold standard when atypical features are present, when there is clinical suspicion of malignancy, or when lesions are in deeper soft tissues [5–7].

Recent advances in evidence-based scoring systems—such as Moran’s septal and size criteria, Nagano’s weighted MRI score, and the ST-RADS classification—have further refined diagnostic pathways, enabling more precise risk stratification and individualized management strategies [8–10]. Although conventional MRI parameters—septal thickness, nodularity, and lesion size—remain fundamental, the integration of dynamic contrast‐enhanced sequences and advanced radiomic features has further refined diagnostic accuracy [11–13]. The combined use of morphological assessment, functional imaging data, and structured reporting enhances malignancy risk stratification and streamlines patient workflows.

Recent evidence-based scoring systems, such as Moran’s septal and size criteria, Nagano’s weighted MRI score, and the ST-RADS classification, have refined diagnostic pathways and improved risk stratification [8–10]. While conventional MRI parameters such as septal thickness, nodularity, and lesion size remain essential, the addition of dynamic contrast-enhanced sequences and radiomic features further enhances diagnostic accuracy. The integration of morphological, functional, and structured reporting approaches contributes to more precise management of lipomatous tumors.

In this review, we synthesize current literature together with our institutional case series to outline a practical framework for MRI assessment of these lesions, highlighting hallmark imaging findings with pathologic correlation and emerging diagnostic approaches that promise to enhance diagnostic confidence and inform therapeutic decision-making.

## Benign fat-containing tumors

### Lipomas

Lipomas are benign, adipose tissue–containing neoplasms that account for approximately half of all soft-tissue tumors, making them the most common subcutaneous neoplasm [14]. They most often present in adults during the fifth to sixth decades of life, although they may occur at any age. On MRI, lipomas are typically well-circumscribed, homogeneously fatty masses; they may display thin septations (< 2 mm) that may enhance after contrast administration [15]. Based on their location relative to the investing fascia of the deep muscles, lipomas are classified as either superficial or deep [14]. Most are encapsulated by a delicate fibrous capsule, but unencapsulated variants also exist. Additionally, regions of nonadipose tissue—such as fibrous stroma, cartilage, calcifications, or myxoid change can introduce internal heterogeneity, sometimes mimicking other soft-tissue neoplasms and complicating the differential diagnosis. Awareness of these imaging characteristics is crucial for accurate lesion characterization, appropriate differential diagnosis, and optimal therapeutic planning [14].

### Superficial lipoma

**Fig. 1:**
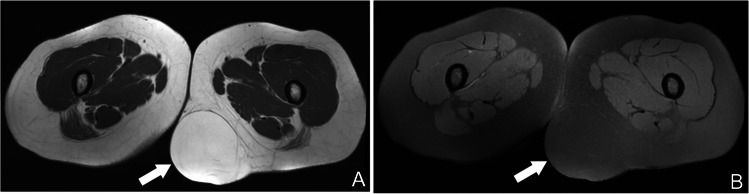
Axial MR images in a 49-year-old woman demonstrate an encapsulated superficial lipoma. (**A**) T1-weighted image and (**B**) fat-suppressed T2-weighted image show a well-circumscribed, homogeneously hyperintense mass within the subcutaneous fat (arrows), identical to subcutaneous fat signal in all sequences. A fine, low-signal-intensity fibrous capsule surrounds the lesion, confirming its encapsulated nature

Superficial lipomas arise within the subcutaneous fat, immediately above the superficial fascia, and typically mirror the signal intensity or attenuation of adjacent adipose tissue, which can make their margins difficult to appreciate on imaging. They may occur as well‐circumscribed, encapsulated masses or as nonencapsulated, infiltrative collections of fat, often referred to as lipomatosis, when a discrete capsule is absent (Fig. 1) [16]. According to current European Society for Medical Oncology (ESMO) guidelines, any homogeneous, superficial adipocytic lesion with a maximal dimension greater than 10 cm should be referred to a dedicated sarcoma center for multidisciplinary evaluation and consideration of image‐guided biopsy [17, 18].

### Intra/intermuscular lipoma

**Fig. 2:**
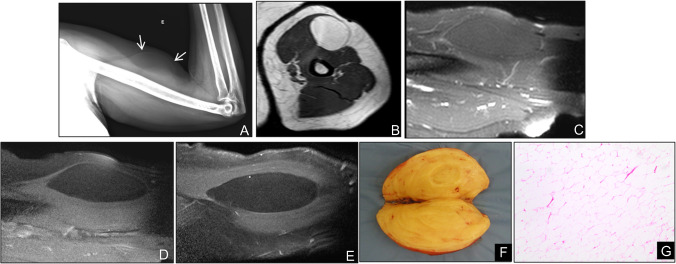
Intramuscular lipoma in a 64-year-old man. (**A**) Radiograph of the right arm demonstrates a well-defined radiolucent soft-tissue mass (arrows). (**B**) Axial T1-weighted and (**C**) fat-suppressed T2-weighted MR images show a homogeneous intramuscular lesion isointense to subcutaneous fat, without evidence of internal non-fatty components (**D**,**E**) Sagittal post-contrast T1-weighted images confirm the absence of significant enhancement. (**F**) Gross pathological specimen reveals a yellow, lobulated mass with thin fibrous septa. (**G**) Photomicrograph (H&E, × 200) shows uniform mature adipocytes without atypia, consistent with benign lipoma

**Fig. 3:**
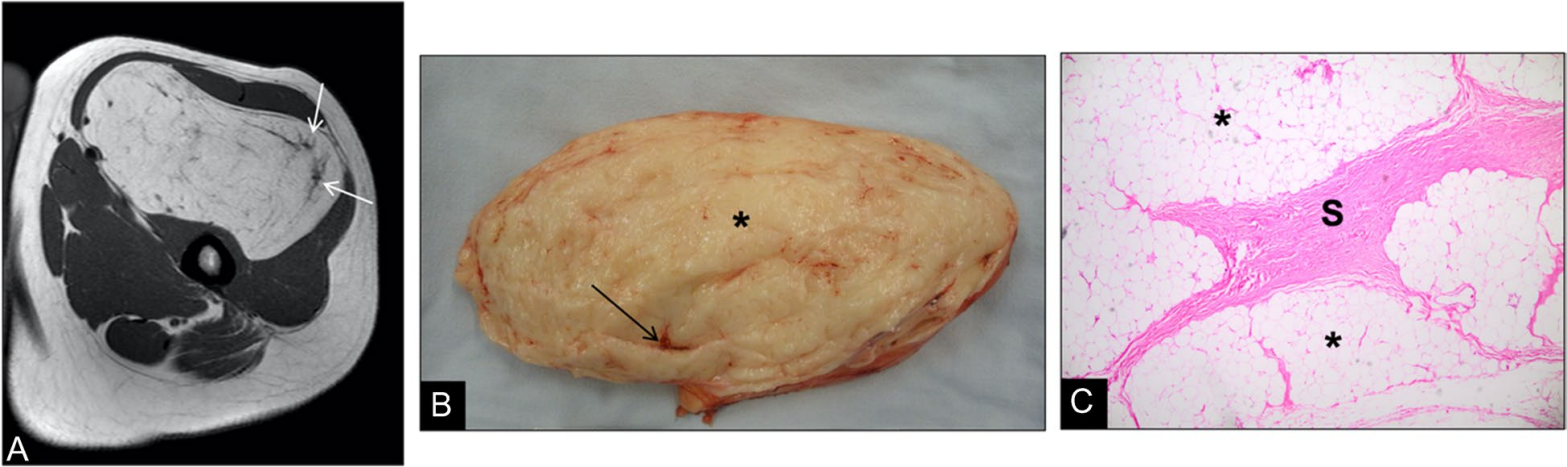
Involutional changes in a longstanding thigh lipoma of a 21-year-old man. (**A**) Axial T1-weighted MR image demonstrates a large intramuscular lesion with signal intensity identical to subcutaneous fat, but containing thick, low-signal septations (arrows) that mimic features of an atypical lipomatous tumor. (**B**) Gross specimen of the resected mass reveals yellow, lobulated adipose tissue (*) traversed by prominent fibrous septa (S). (**C**) Photomicrograph (H&E, × 100) shows uniform mature adipocytes (*) separated by thick collagenous septa (S) without cytologic atypia, confirming benign involutional changes

Intramuscular and intermuscular lipomas occur deep to the investing fascia, within or between muscle bundles. On imaging, they typically appear as well-defined, homogeneously fatty masses without thick septations (> 2 mm), nodular soft-tissue components, or non-adipose areas—features that, when present, raise concern for more aggressive pathology (Fig. 2). Whenever a deep adipocytic lesion deviates from these benign criteria, by exceeding 5 cm in maximal diameter, demonstrating internal heterogeneity, or showing suspicious septa or nodularity, lipoma variants, involutional changes, or, critically, liposarcoma must be considered (Fig. 3). In such cases, and per *ESMO* recommendations, referral to a dedicated sarcoma center for multidisciplinary assessment and image-guided biopsy is advised for any deep-seated adipocytic mass of the trunk or extremities [17, 18].

### Lipoma variants

Lipoma variants encompass a heterogeneous group of benign adipocytic neoplasms whose imaging appearances often diverge from those of ordinary lipomas and may closely mimic well‐differentiated liposarcomas. According to the WHO classification, lipomas with a component of normal-appearing bone, cartilage, or fibrous connective tissue may be designated as osteolipomas, chondrolipomas, or fibrolipomas, respectively. However, these lesions are still considered as lipoma subtypes, included within the same nosological category as the purely adipocytic conventional lipoma. In contrast, there are several benign adipocytic neoplasms that are considered independent entities, including lipoblastoma, hibernoma, chondroid lipoma, angiolipoma, spindle cell/pleomorphic lipoma, atypical spindle cell/pleomorphic lipomatous tumor, and myolipoma [19]. Each entity exhibits unique histologic and clinical features, such as multilobulated architecture in lipoblastoma, brown fat attenuation in hibernoma, or prominent vascularity in angiolipoma, that can translate into atypical signal characteristics, septal thickening, or nodularity on MRI. Given this overlap with malignant adipocytic tumors, biopsy or complete excision is often warranted to establish a definitive diagnosis and guide management. Recognizing these variants in the differential diagnosis of any fat‐containing soft‐tissue mass is essential to direct appropriate multidisciplinary evaluation and treatment.

### Lipoblastoma and diffuse lipoblastoma

**Fig. 4:**
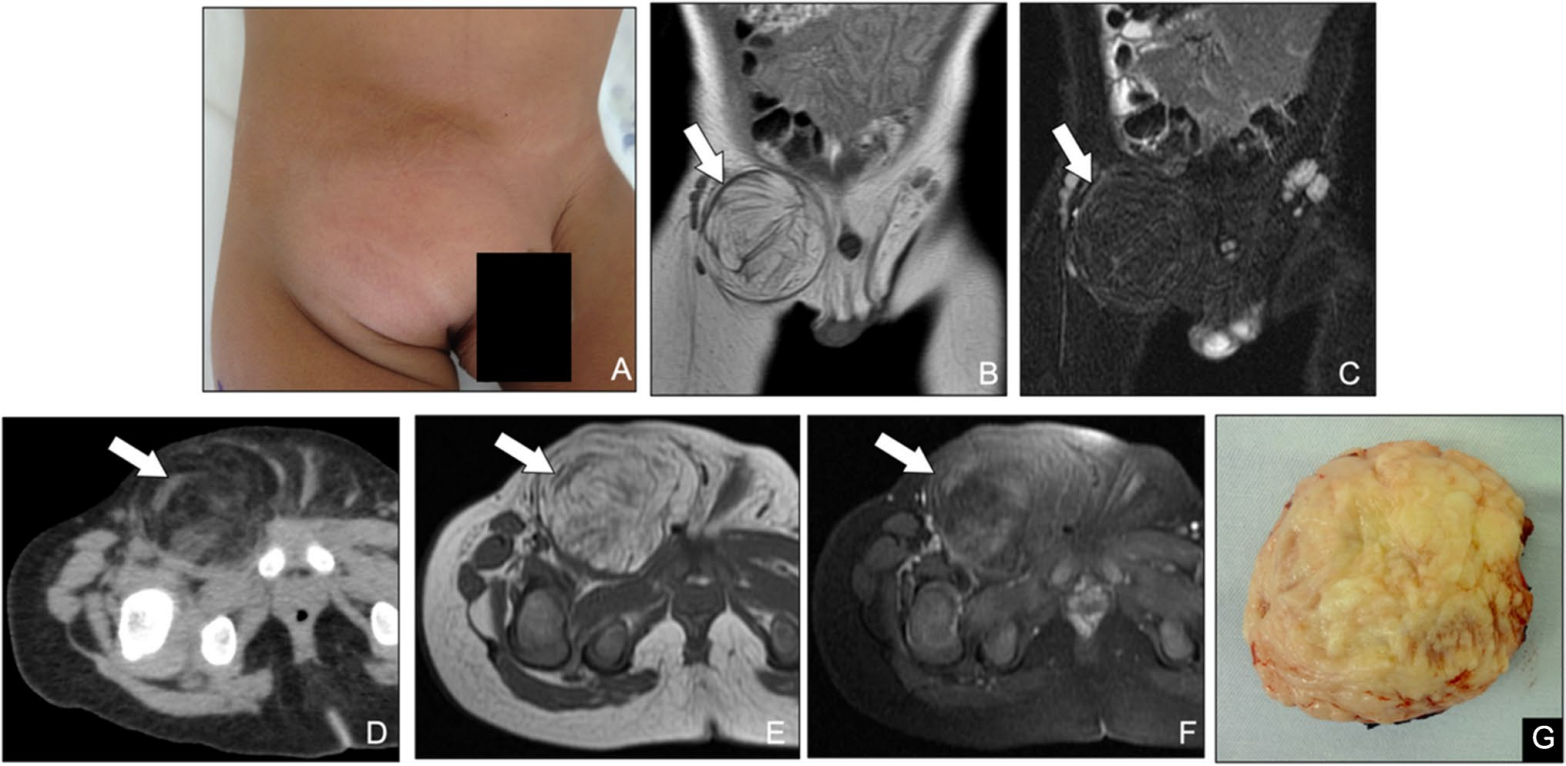
Lipoblastoma of the right inguinal region in a 1-year-old boy. (**A**) Clinical photograph demonstrates a palpable, well-circumscribed inguinal mass. (**B**) Coronal T1-weighted MR image and (**C**) fat-suppressed T2-weighted MR image show a predominantly fatty lesion with thick, irregular septa (arrows). (**D**) Axial CT image confirms a low-attenuation fatty mass with internal septations. (**E**) Axial T1-weighted and (**F**) post-contrast T1-weighted MR images further delineate the extent of the lesion and enhancement of the fibrous septa (arrows)(**G**) Gross specimen photograph reveals lobulated adipose tumor nodules separated by firm fibrous septa

**Fig. 5:**
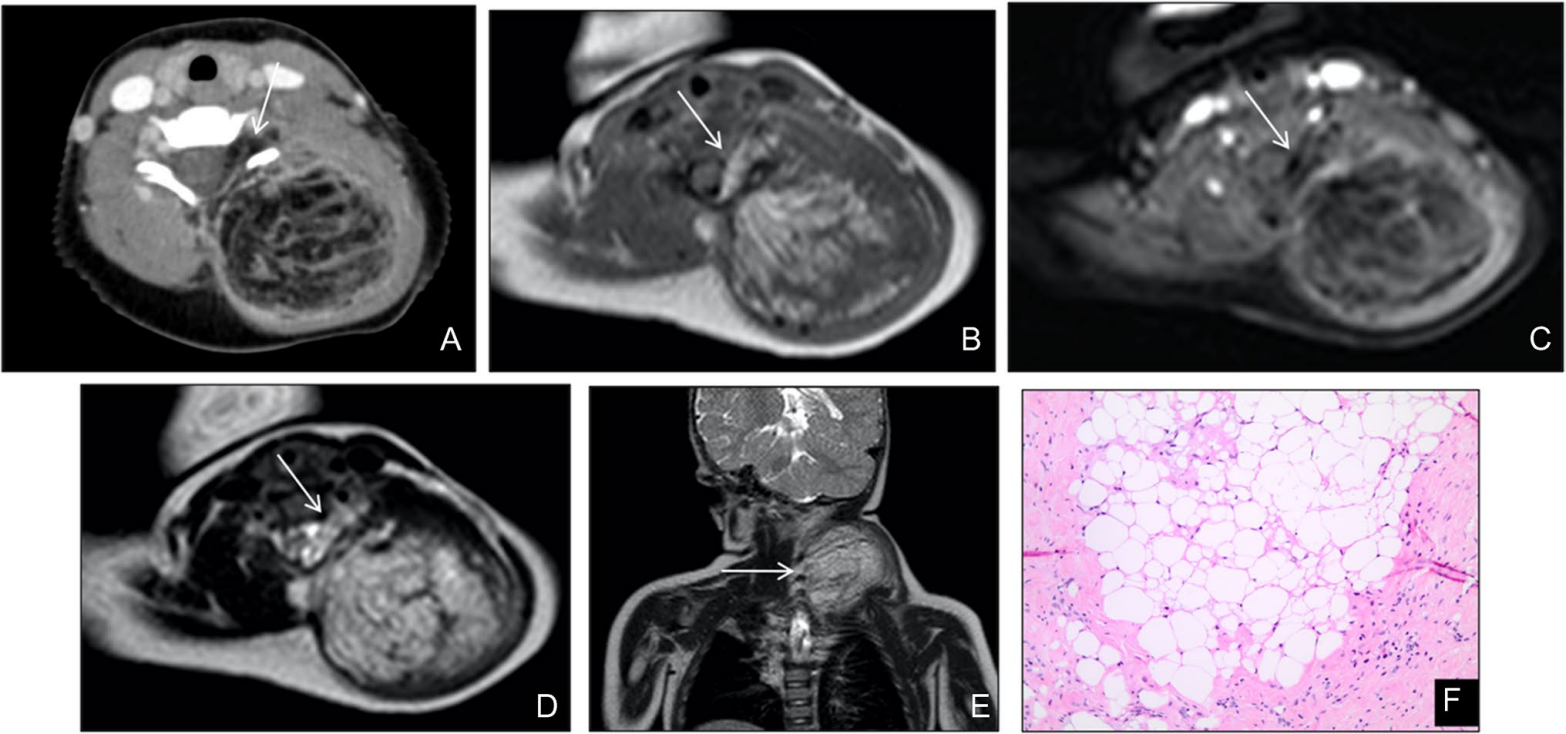
Infiltrative cervical lipomatous lesion. (**A**) Contrast-enhanced CT shows a fat-attenuation mass in the left neck containing streaky higher-density areas and extending through the neural foramina, causing bony enlargement and rightward displacement of the dural sac (arrows). Axial T1-weighted image (**B**) and axial (**D**) and coronal (**E**) T2-weighted MR images confirm a predominantly fatty lesion with interspersed thick septa (**C**) Axial post-contrast T1-weighted MR images demonstrate heterogeneous enhancement of the thick fibrous septations. (**F**) Photomicrograph (H&E, × 200) reveals mature adipocytes arranged in lobules separated by fibrous septa, with scattered spindle cells, lipoblasts, and a myxoid stromal background

Lipoblastomas are rare, benign adipocytic tumors that arise almost exclusively in infants and young children, with a male predominance of approximately 2–3:1. They most often involve the extremities and present in two forms: a well-circumscribed variant (lipoblastoma; Fig. 4) or an infiltrative, multicentric pattern known as diffuse lipoblastoma (Fig. 5) [20–22]. Histologically, these lesions contain immature, embryonal adipocytes that may, over time, differentiate into mature fat, occasionally resulting in spontaneous maturation to a conventional lipoma. Maturation occurs predominantly in the center of the tumor lobules, while immature tissue persists at the edges, consisting of lipoblasts in various stages of maturation, often within a myxoid matrix. These immature portions can be very similar histologically to myxoid liposarcoma, but in this tumor, the maturation pattern is reversed, that is, the more mature adipose cells predominate at the periphery of the lobules [19, 23]. On CT and MRI, lipoblastomas typically appear as predominantly fatty masses; however, their immature myxoid or cellular regions can produce nonspecific signal characteristics that overlap with those of liposarcomas, a malignancy exceedingly uncommon in the pediatric population [24]. Because imaging alone cannot reliably distinguish lipoblastoma from well-differentiated liposarcoma, correlation with patient age and prompt histopathologic confirmation are essential. The treatment of choice is complete local excision, aiming for clear margins while preserving surrounding structures to optimize functional outcomes and minimize recurrence [25].

### Chondroid lipoma

**Fig. 6:**
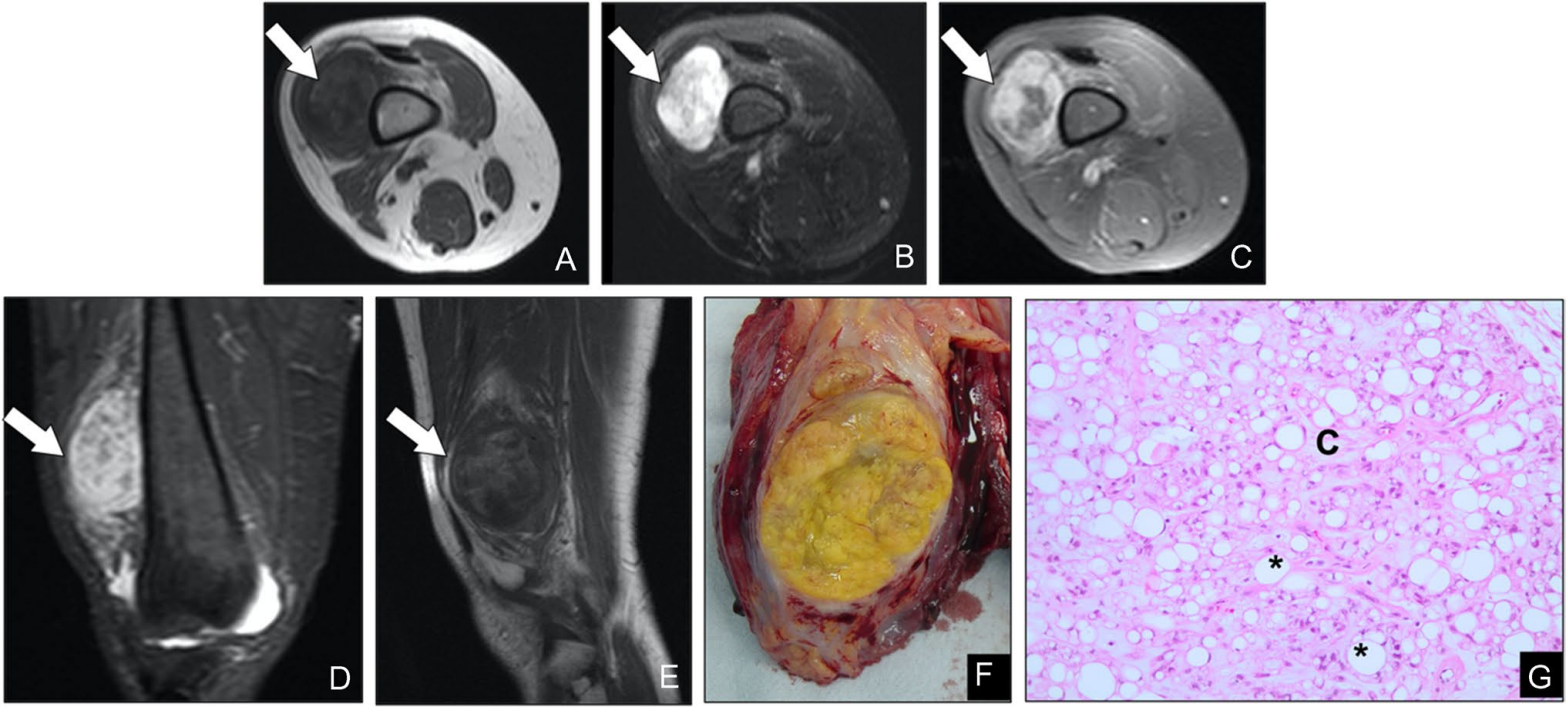
Chondroid lipoma in a 51-year-old woman presenting with a slow-growing thigh mass. Axial (**A**) and sagittal (**E**) T1-weighted MR images show a well-defined heterogeneous lesion with overall low signal intensity and a small hyperintense focus indicating intralesional fat (arrows). Axial (**B**) and coronal (**D**) fat-suppressed T2-weighted images reveal lobulated areas of high signal consistent with a myxoid–chondroid matrix. (**C**) Axial post-contrast fat-saturated T1-weighted image demonstrates heterogeneous enhancement throughout the lesion. (**G**) Gross specimen exhibits a gelatinous, lobulated cut surface reflecting the myxoid components.(**H**) Photomicrograph (H&E, × 200) demonstrates mature adipocytes (*) and multivacuolated lipoblast-like cells embedded within a chondromyxoid matrix (**C**). Although the imaging appearance may mimic myxoid liposarcoma or extraskeletal myxoid chondrosarcoma, the absence of aggressive imaging features and the histologic demonstration of a chondromyxoid matrix without true cartilaginous differentiation support the diagnosis of chondroid lipoma

Chondroid lipomas are rare, benign, slow-growing adipocytic tumors that most often present in women during the third to fourth decades of life [26]. They typically arise in the extremities or limb girdles and are histologically characterized by nests of lipoblasts embedded within a myxoid or hyalinized chondroid matrix, interspersed with variable amounts of mature fat [26]. On MRI, these lesions appear as well-defined, lobulated masses with areas of T2-hyperintense, fluid-like signal and show variable postcontrast enhancement (Fig. 6) [27, 28]. Radiographs may occasionally reveal internal calcifications. Because chondroid lipomas can mimic myxoid liposarcoma or extraskeletal myxoid chondrosarcoma on imaging, histopathologic confirmation, either by image-guided biopsy or excisional sampling is recommended to establish a definitive diagnosis and guide appropriate management [29].

### Angiolipoma

Angiolipomas are benign, encapsulated subcutaneous tumors composed of mature adipocytes interspersed with small, thin-walled blood vessels. These proliferated capillaries commonly exhibit characteristic fibrin microthrombi and predominate at the tumor periphery [19]. Angiolipomas most commonly present as small, often multifocal, tender nodules in adolescents and young adults, particularly along the forearm, but are rare in children [30]. Clinically, patients frequently report pain on palpation. Ultrasonography is the modality of choice for initial evaluation, typically revealing an ovoid, heterogeneous, hyperechoic mass with internal vascularity on Doppler imaging, features that reliably distinguish angiolipoma from the more homogeneous, isoechoic, spindle-shaped lipoma, which lacks intrinsic blood flow [31, 32]. On CT and MRI, angiolipomas appear as fat-containing lesions “dirtied” by their vascular channels; postcontrast studies characteristically demonstrate enhancement of the nonadipose components, permitting a confident imaging diagnosis when correlated with clinical presentation [30].

### Hibernoma

**Fig. 7:**
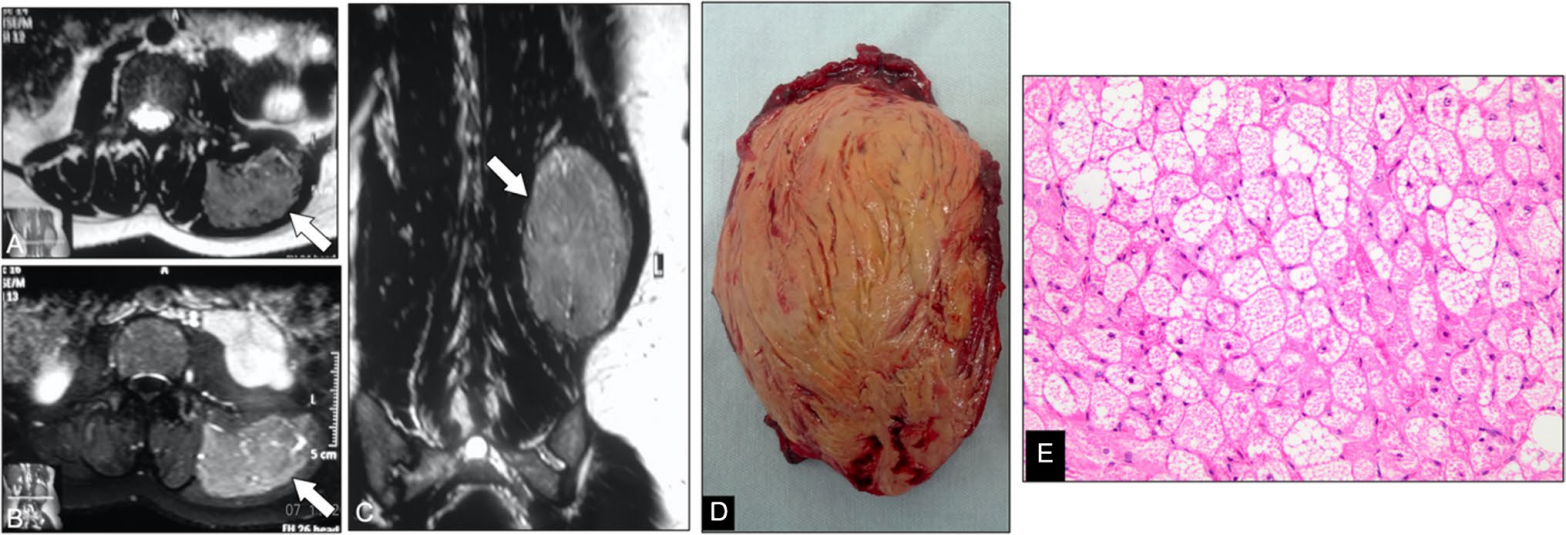
Intramuscular hibernoma in a 35-year-old woman. (**A**, **B**) Axial and (**C**) coronal T2-weighted MR images reveal a well-defined mass within the left paravertebral musculature that is slightly hypointense compared with subcutaneous fat (arrows). (**B**) Axial fat-suppressed, post-contrast T1-weighted image shows heterogeneous enhancement. (**D**) Gross specimen photograph demonstrates the characteristic yellow–brown cut surface with prominent central vessels. (**E**) Photomicrograph (H&E, × 200) displays polygonal eosinophilic “brown fat” cells with granular cytoplasm intermixed with univacuolated adipocytes, confirming hibernoma

Hibernomas are rare, benign soft-tissue neoplasms composed of brown fat cells, most often presenting in adults during the third to fifth decades of life and arising predominantly in the proximal thigh, shoulder, back, or neck [19]. Four histologic variants are recognized: typical (the most common), lipoma-like, myxoid, and spindle cell, each defined by the relative proportion of multivacuolated brown fat cells, univacuolated adipocytes, myxoid stroma, or spindle cell elements. On MRI, hibernomas appear as well-circumscribed, fat-containing masses with signal intensity slightly lower than subcutaneous fat on T1-weighted sequences and incomplete suppression on STIR or fat-saturated T2-weighted images (Fig. 7). Internal thin septations and prominent serpentine or branching vessels producing flow voids are characteristic. However, in some cases hibernomas may mimic atypical lipomatous tumors, and histopathologic confirmation is then required for definitive diagnosis. As a result of their active role in thermogenesis, hibernomas usually demonstrate markedly increased FDG uptake on PET [33–35]. Because hibernomas are benign and do not metastasize, complete surgical excision with negative margins is curative. Local recurrence is exceedingly rare and almost always attributable to incomplete resection, underscoring the importance of a thorough surgical approach [36].

### Myolipoma

Myolipoma is a rare benign neoplasm composed of mature adipose tissue and smooth muscle cells, with a strong female predominance and peak incidence in the fifth to sixth decades of life [19]. Most lesions arise in the abdominal cavity, retroperitoneum, or inguinal regions, although cases in the trunk, extremities, and subcutaneous tissues have been reported [5, 14]. Due to their deep location, tumors are usually large at presentation, often encapsulated, and range from 10–25 cm in size [14].

At gross pathologic examination, these large tumors are usually completely or at least partially encapsulated with a yellow to white appearance. Histologically, myolipomas demonstrate an admixture of smooth muscle and adipose tissue, typically with smooth muscle predominance (2:1), imparting a characteristic “sieve-like” appearance. Immunohistochemistry confirms strong positivity for smooth muscle actin and desmin [14, 19].

On imaging, myolipomas are heterogeneous, reflecting intermixed fat and soft-tissue components. The adipose tissue appears as high T1 and T2 signal that suppresses with fat saturation, whereas the smooth muscle components demonstrate intermediate T1 and intermediate-to-high T2 signal intensity, sometimes with coarse calcifications [5, 14]. Because of these nonspecific features, differentiation from well-differentiated liposarcoma is often not possible on imaging alone [14, 17]. Complete surgical excision is curative, and no cases of recurrence, metastasis, or malignant transformation have been reported [14, 19].

### Spindle cell/pleomorphic lipoma

Spindle cell/pleomorphic lipoma is a benign adipocytic neoplasm that typically arises in middle-aged to older adults, with a strong male predominance. It most commonly involves the posterior neck, shoulder, and upper back, although lesions may also occur in the trunk and extremities. In the 2020 WHO classification, spindle cell lipoma and pleomorphic lipoma are regarded as part of a single disease spectrum, reflecting shared histologic, genetic, and clinical features [19].

Histologically, these tumors are characterized by a variable admixture of mature adipocytes, bland spindle cells arranged in short fascicles or a “school-of-fish” pattern, rope-like collagen bundles, and, in pleomorphic variants, multinucleated floret-like giant cells. Despite their pleomorphic appearance, mitotic activity is minimal and biologic behavior is indolent. Recurrent deletions involving chromosomes 13q and/or 16q are characteristic, and immunohistochemistry typically demonstrates CD34 positivity, with absence of MDM2 and CDK4 amplification, helping to distinguish these lesions from atypical lipomatous tumor/well-differentiated liposarcoma [19, 23, 37].

**Fig. 8:**
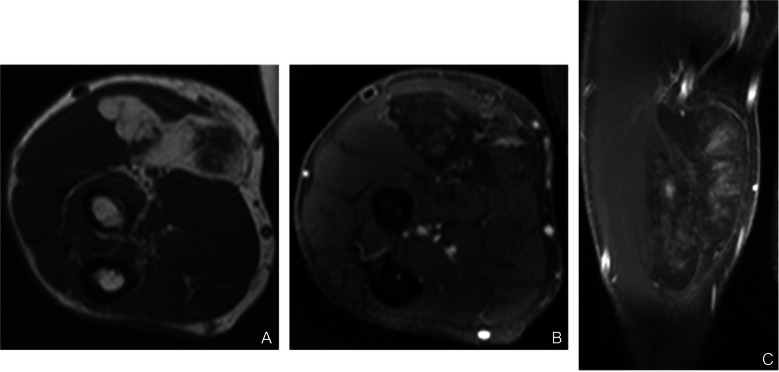
Spindle cell/pleomorphic lipoma of the right forearm. Axial T1-weighted image (**A**) shows a well-circumscribed intermuscular mass in the anterolateral region, with heterogeneous signal intensity, predominantly fatty, and containing non-adipose components. Axial fat-suppressed T2-weighted (**B**) and coronal post-contrast fat-suppressed T1-weighted (**C**) images demonstrate suppression of the fatty areas and relative T2 hyperintensity of the non-adipose component, which shows post-contrast enhancement. Although this appearance may mimic atypical lipomatous tumor, the typical anatomic distribution, limited lesion size, absence of thick septa or nodular soft-tissue components, and benign histologic features favor the diagnosis of spindle cell/pleomorphic lipoma

On MRI, spindle cell/pleomorphic lipomas often demonstrate variable fat content, resulting in a heterogeneous appearance that may deviate from that of a conventional lipoma. Non-adipose components commonly appear isointense to muscle on T1-weighted images and show variable signal intensity on T2-weighted sequences, depending on the relative proportions of spindle cells, collagen, and myxoid stroma. Contrast-enhanced MRI typically reveals mild to moderate enhancement of the non-fatty componentes (Fig. 8). These imaging features can closely mimic atypical lipomatous tumor, particularly when thickened septa or nodular non-adipose areas are present, representing a frequent diagnostic pitfall.

Recognition of the typical anatomic distribution, limited lesion size, and lack of aggressive imaging features, such as infiltrative margins, large nodular soft-tissue components, or marked enhancement, may suggest the diagnosis. However, due to substantial overlap with other adipocytic neoplasms, imaging findings alone are insufficient for definitive characterization, and histopathologic confirmation is often required. Complete local excision is curative, and recurrence is uncommon, with no metastatic potential reported.

### Atypical spindle cell/pleomorphic lipomatous tumor

Atypical spindle cell/pleomorphic lipomatous tumor (ASPLT) is a recently recognized benign adipocytic neoplasm included in the 2020 WHO classification [19]. It was previously named spindle cell liposarcoma and considered a rare subtype of well-differentiated liposarcoma/atypical lipomatous tumor. However, it has become clear that it is genetically distinct from these neoplasms, lacking the characteristic amplification of *MDM2*, and that it poses no known risk of dedifferentiation [19, 23]. It typically occurs in middle-aged adults with a slight male predominance and most commonly involves the extremities, limb girdles, or trunk.

Histologically, ASPLTs are composed of variable proportions of bland to mildly atypical spindle cells, pleomorphic multinucleated giant cells, mature adipocytes, and scattered lipoblasts within a fibrous to myxoid stroma [14, 18]. The spindle cells tend to be oriented in bundles, similar to low-grade fibrosarcoma, or have an appearance reminiscent of neural neoplasia. These features help differentiate it from ALT/WDLPS, in addition to the absence of immunohistochemical coexpression of MDM2 and CDK4 and the frequent CD34 positivity [19, 23, 37].

On MRI, these tumors usually appear as poorly circumscribed, heterogeneous masses with intermixed fat and nonadipose components. Signal intensity is variable, reflecting the fibrous, myxoid, and lipomatous elements, with mild to moderate enhancement after contrast. Because of overlapping features with other adipocytic tumors, imaging findings alone are not diagnostic [17].

Despite their atypical histology, ASPLTs follow an indolent clinical course. Complete surgical excision is the treatment of choice, with local recurrence possible but no metastasis described [19].

### Lipoma arborescens

**Fig. 9:**
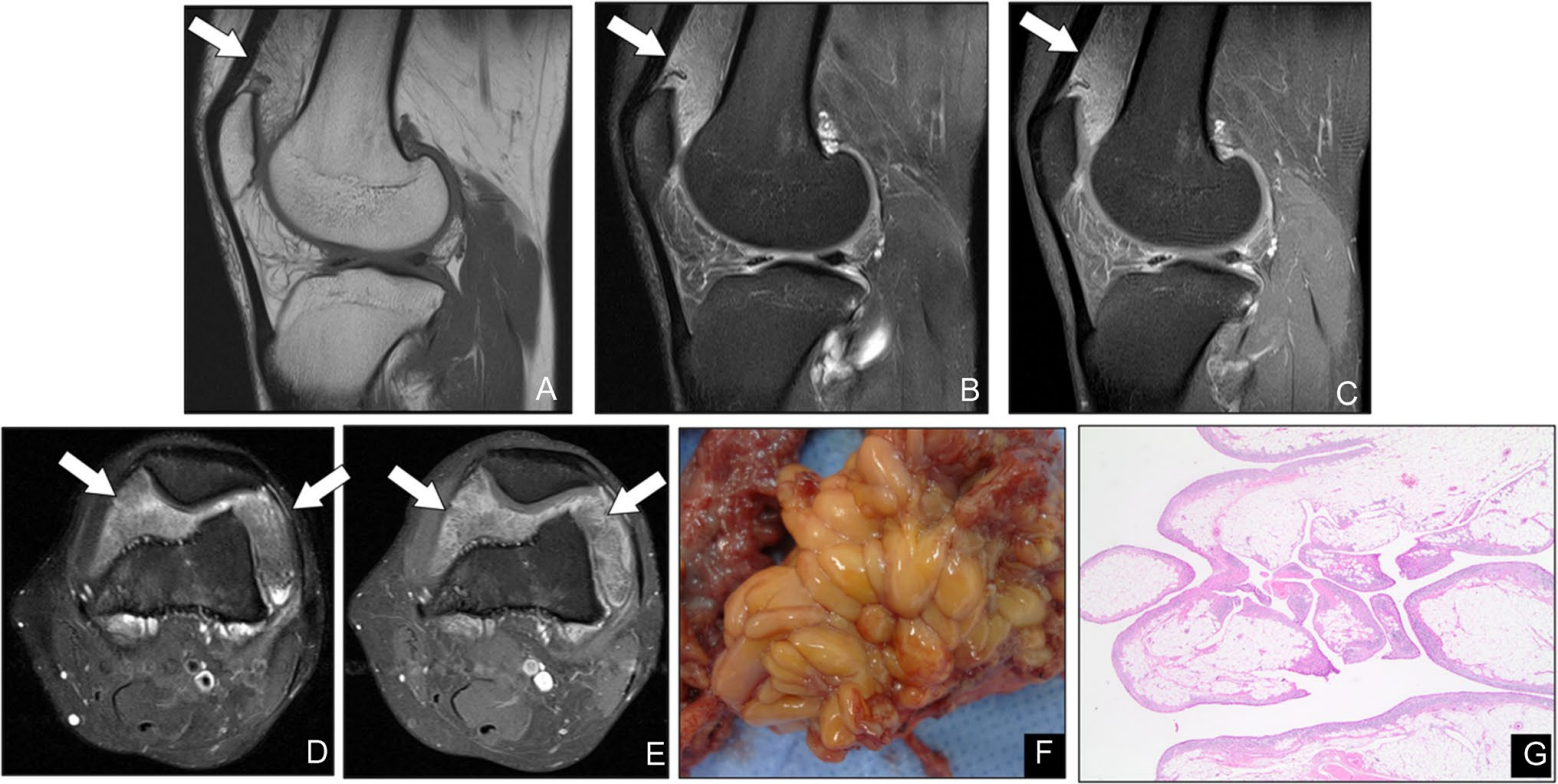
Lipoma arborescens in a 26-year-old man presenting with a swollen knee. (**A**–**C**) Sagittal T1 (**A**), sagittal T2 fat-suppressed (**B**), and sagittal T1 fat-suppressed postcontrast (**C**) images; and (**D**, **E**) axial T2 fat-suppressed (**D**) and axial T1 fat-suppressed postcontrast (**E**) images demonstrate frondlike synovial villi within the suprapatellar bursa (arrows), all exhibiting signal intensity identical to that of subcutaneous fat. (**F**) Gross specimen of resected synovium shows yellow, villous projections consistent with fatty infiltration. (**G**) Photomicrograph (**H**&**E**, × 100) reveals hypertrophied synovial villi with a central core of mature adipose tissue, confirming the diagnosis of lipoma arborescens

Lipoma arborescens is a distinctive, nonneoplastic process characterized by frondlike proliferation of mature adipocytes within the synovial subintimal connective tissue, most frequently involving the suprapatellar recess of the knee. Although its precise etiology remains unclear, it is not listed in the WHO classification of neoplasms [19], and it is now regarded as a reactive phenomenon linked to chronic joint irritation — commonly seen in degenerative osteoarthritis, rheumatoid arthritis, or after prior trauma [38, 39]. Histologically, the synovium’s subintimal layer is replaced by lobules of mature fat cells, imparting a villous or “tree‐like” appearance [40]. On MRI, lipoma arborescens presents as a frondiform mass of high T1 and T2 signal that suppresses on fat‐saturated sequences, often accompanied by joint effusion and synovial hypertrophy (Fig. 9). These characteristic imaging features, together with its predilection for the suprapatellar pouch, allow for confident, noninvasive diagnosis. Arthroscopic synovectomy or open excision of the fatty villi provides both symptom relief and definitive treatment, with low rates of recurrence.

### Lipomatosis of nerve

**Fig. 10:**
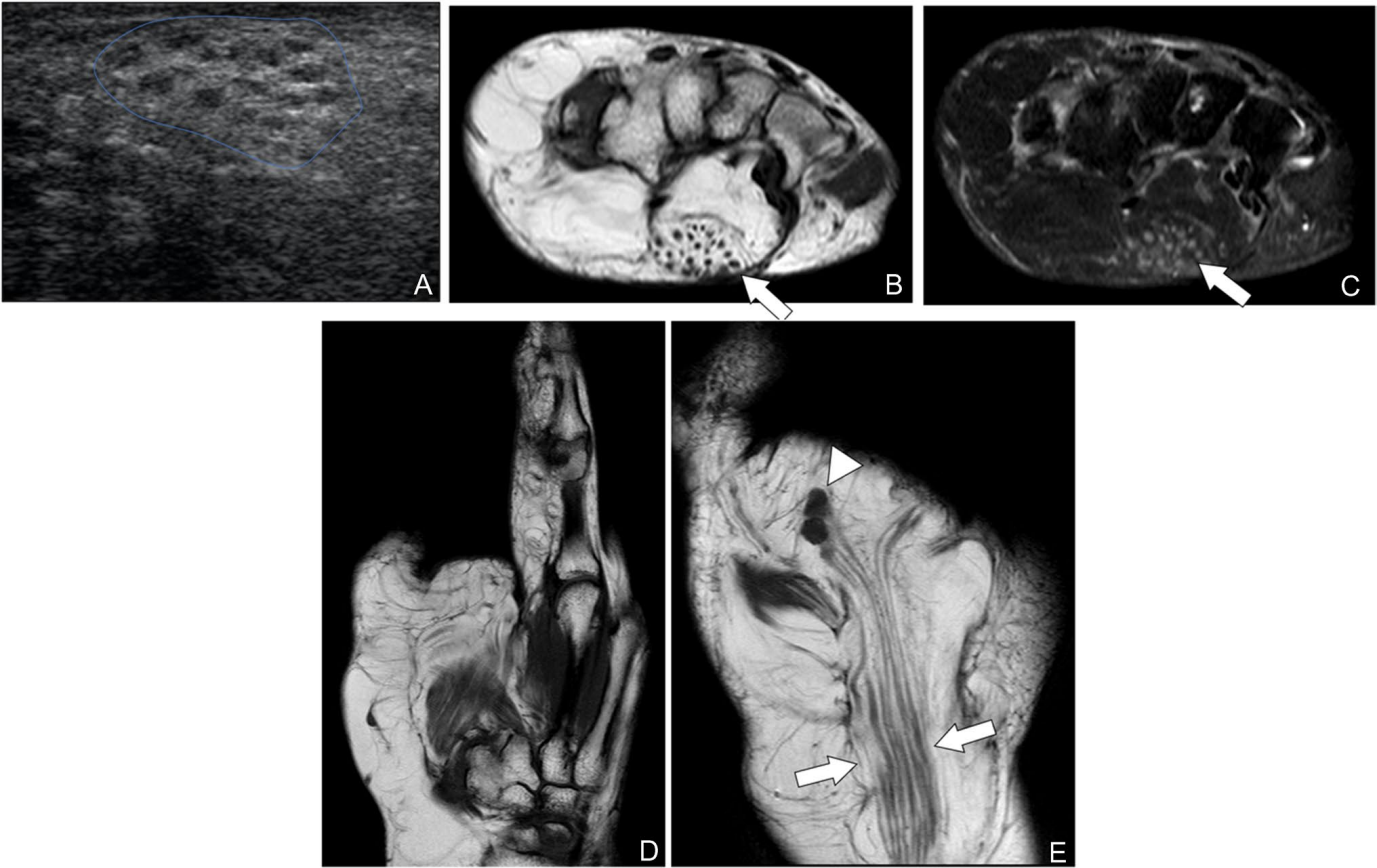
Macrodystrophia lipomatosa with median nerve lipomatosis in a 25-year-old man. (**A**) Ultrasonography of the forearm demonstrates an enlarged median nerve with tubular hypoechoic fascicles separated by thickened hyperechoic fat (arrows). (**B**) Axial T1-weighted MR image confirms nerve enlargement and fatty infiltration along the fascicular bundles. (**C**) Axial fat-suppressed T2-weighted image shows persistent high signal of the interspersed fat without intrinsic nerve edema. (**D**) Coronal T1-weighted MRI illustrates prior amputation of the second digit, with stump neuroma formation at the distal median nerve (arrowhead). (**E**) Coronal T1-weighted MR image further characterizes the extent of nerve lipomatosis and associated soft-tissue overgrowth

**Lipomatosis of nerve** is a rare, benign condition marked by fibrofatty overgrowth within peripheral nerves, leading to fusiform enlargement and splaying of the nerve fascicles. It most commonly affects the upper extremity, especially the median nerve [41]. On MRI, the affected nerve demonstrates pronounced, diffuse enlargement with characteristic T1-hyperintense fat interdigitating between the normally T1-hypointense nerve fascicles, creating a “coaxial cable” or “spaghetti” appearance (Fig. 10). The nature of the lesion is not clearly defined; it is generally regarded as a hamartoma, but often shows progressive behavior resembling that of a neoplasm [19]. Lipomatosis of nerve is often associated with macrodystrophia lipomatosa, in which there is overgrowth of bone and soft tissues in the same nerve distribution [42]. Because the imaging findings are pathognomonic, biopsy is generally avoided to prevent iatrogenic neurologic injury [43]. Recognition of these distinctive imaging features is therefore critical to establish the diagnosis noninvasively and to guide appropriate, conservative management.

### Intermediate fat-containing tumors: Well-differentiated liposarcoma/atypical lipomatous tumor

Atypical lipomatous tumor (ALT) and well-differentiated liposarcoma (WDLPS) are synonymous entities that share similar morphologic and genetic profiles. The term “atypical lipomatous tumor” is used when the lesion is surgically resectable (e.g., extremities), whereas “well-differentiated liposarcoma” applies to tumors in locations where complete excision with wide margins is challenging (e.g., retroperitoneum) [19, 44]. These tumors comprise approximately 50% of liposarcomas and characteristically contain ≥ 75% adipose tissue. Common sites for liposarcomas include the extremities, retroperitoneum, paratesticular regions, and mediastinum [19].

Histologically, ALT/WDLPS is composed mainly of mature adipocytes and non-lipogenic spindle cells with fibroblastic morphology, arranged within irregular fibrous septa or broader fibrous areas. The relative proportion of these elements defines the lipoma-like (adipocytic, most common) and sclerosing variants, while the inflammatory variant is rare and shows a dense inflammatory infiltrate [19, 23, 37]. The defining feature distinguishing ALT/WDLPS from conventional lipoma or fibrolipoma is the presence of atypical adipocytes or non-lipogenic cells, characterized by nuclear enlargement, hyperchromasia, and pleomorphism. Atypical cells may be scarce or numerous. Lipoblasts—immature adipocytes with hyperchromatic nuclei indented by one or more lipid vacuoles—may also occur [19, 23, 37]. Rare cases exhibit heterologous differentiation, such as osteosarcomatous or leiomyosarcomatous elements, always low grade and not indicative of dedifferentiation. Smooth muscle components may also appear as atypical cells in the walls of intratumoral vessels [23, 37].

**Fig. 11:**
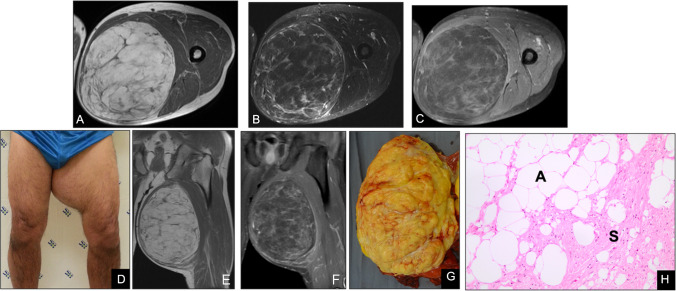
Atypical lipomatous tumor of the thigh in a 61-year-old man (**D**). (**A**) Axial T1-weighted and (**B**) fat-suppressed T2-weighted MR images depict a large, well-circumscribed mass with predominantly fat signal and thick, low-signal septations. (**C**) Post-contrast fat-suppressed T1-weighted image shows enhancement of the internal septa. (**E**) Coronal T1-weighted MR image further illustrates the lesion’s heterogeneous appearance and deep intramuscular location. (**F**) Additional post-contrast sequence confirm robust septal enhancement without nodular nonadipose components. (**G**) Gross specimen photograph reveals a yellow lobulated mass with conspicuous fibrous septa. (**H**) Photomicrograph (H&E, × 200) demonstrates mature adipocytes and thick collagenous septa with atypical cells (S), consistent with an atypical lipomatous tumor. While involutional or fibrous lipomas may also exhibit internal septations, the presence of prominent enhancing septa, nodular soft-tissue elements, and reduced internal fat fraction raises suspicion for atypical lipomatous tumor/well-differentiated liposarcoma and warrants histopathologic confirmation

On MRI, ALT/WDLPS present as predominantly fatty masses with thick (> 2 mm), irregular septa, nodular non-adipose regions, and marked septal enhancement (Fig. 11). Although these findings can overlap with lipoma variants or involuted lipomas—creating diagnostic pitfalls—several features favor a diagnosis of ALT/WDLPS: lesion size > 10 cm, prominent or nodular septations, reduced internal fat fraction, globular non-adipose nodules, conspicuous T2-hyperintense foci, avid contrast uptake, and an intramuscular location. Despite their low metastatic potential, ALT/WDLPS are locally aggressive, exhibit high recurrence rates, and carry a risk of dedifferentiation into high-grade sarcoma. Therefore, histopathologic confirmation is mandatory, and management typically involves wide local excision to minimize the risk of relapse [44–47].

## Malignant fat-containing tumors

### Dedifferentiated liposarcoma

In the classic definition, dedifferentiated liposarcoma (DDLPS) arises when a well-differentiated liposarcoma undergoes transition into a high-grade, nonlipogenic sarcomatous component, most commonly de novo (~ 90%) but also as progression (~ 10%) [44, 48].

Histologically, the high-grade sarcomatous component of DDLPS usually lacks specific differentiation and resembles undifferentiated pleomorphic sarcoma (formerly malignant fibrous histiocytoma), fibrosarcoma, or myxofibrosarcoma. About 10% of cases show heterologous differentiation (osteosarcomatous, chondrosarcomatous, leiomyosarcomatous, rhabdomyosarcomatous) or homologous features similar to pleomorphic or myxoid liposarcoma [19, 23, 37].

The concept of low-grade dedifferentiation remains controversial, largely due to limited interobserver agreement, although it is recognized in the 2020 WHO classification. Some ALT/WDLPS may exhibit moderately hypercellular non-lipogenic areas consistent with low-grade dedifferentiation [49], particularly when associated with ≥ 5 mitoses per 10 high-power fields, a criterion not universally accepted [37, 50].

Although classical diagnostic criteria require identification of a well-differentiated component, diagnosis of DDLPS based solely on detection of characteristic genetic alterations in a high-grade sarcoma is currently accepted, since the well-differentiated areas may be absent in the sample or obliterated by the dedifferentiated component [19, 51].

**Fig. 12:**
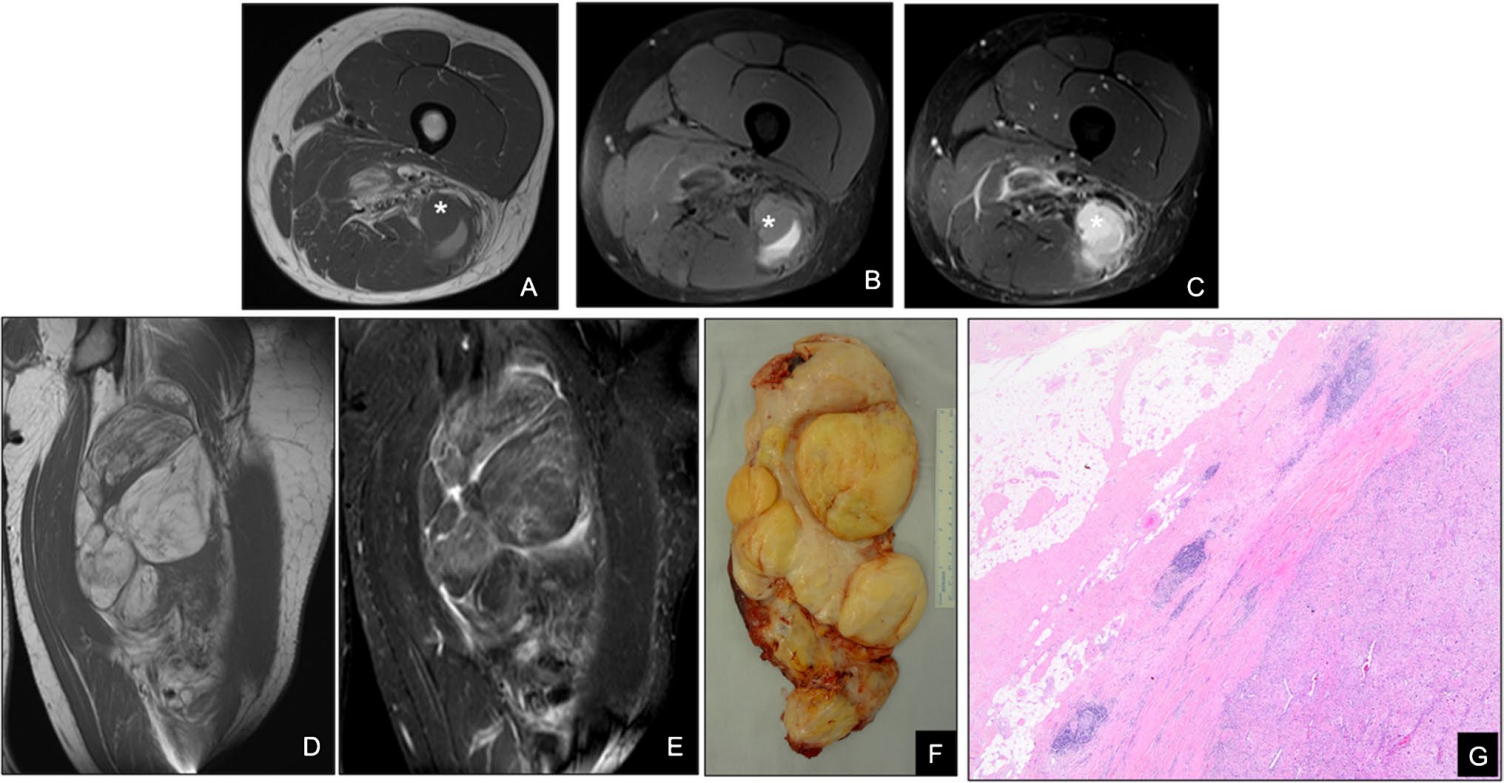
Dedifferentiated liposarcoma of the thigh in a 42-year-old man. (**A**) Axial T1-weighted MR image and (**B**) fat-suppressed T2-weighted MR image reveal a large intramuscular mass containing both mature fat lobules and a nonadipose high-grade component (*). (**C**) Post-contrast fat-suppressed T1-weighted image demonstrates intense enhancement of the nonfatty region, consistent with dedifferentiation. (**D**) Coronal T1-weighted and (**E**) coronal STIR images further demonstrate signal intensity heterogeneity within the lesion, which extends through the muscle belly. (**F**) Gross specimen shows yellow, lobulated adipose tissue interspersed with firm, whitish nonlipomatous areas. (**G**) Photomicrograph (**H**&E, × 100) illustrates juxtaposition of well-differentiated adipocytes (upper left) and high-grade spindle cell sarcomatous tissue (lower right), confirming dedifferentiated liposarcoma. Although complex lipomatous tumors may occasionally display heterogeneous features, the presence of a distinct solid enhancing component adjacent to a fatty mass is highly suggestive of dedifferentiation and supports the diagnosis of dedifferentiated liposarcoma

On MRI, DDLPS typically shows a biphasic pattern: areas of fatty tissue resembling well-differentiated liposarcoma adjacent to irregular, solid masses with intermediate-to-low T1 signal and heterogeneous T2 intensity, often with intense nodular enhancement on contrast-enhanced sequences (Fig. 12) [52].

Kawaguchi et al. reported that DDLPS consistently contains non-adipose components and is more likely to demonstrate tail sign, necrosis, ill-defined margins, and peritumoral edema compared to atypical lipomatous tumors [53]. Diffusion-weighted imaging and chemical shift techniques may provide additional value, and thick septations or nodules ≥ 1 cm are important discriminators [13]. Management requires wide surgical resection, usually combined with adjuvant radiotherapy to reduce recurrence risk.

### Myxoid liposarcoma

Myxoid liposarcoma represents 20–50% of all liposarcomas, making it the second most prevalent subtype of adipocytic neoplasms [54]. It characteristically arises in adults during the fourth to fifth decades of life, although it may also affect younger patients, and typically harbours less than 25% macroscopic fat [55]. Approximately 75–80% of tumours occur in the lower extremities.

Histologically, myxoid liposarcoma is defined by abundant myxoid stroma and a delicate, branching “chicken-wire” capillary network. The neoplastic component consists of relatively small, monomorphic spindle to oval non-lipogenic cells, together with adipocytes at different stages of maturation, ranging from multivacuolated lipoblasts and univacuolated “signet-ring” lipoblasts—the most characteristic cell type—to mature adipocytes [19, 23, 37].

Areas of round cells may also be present, showing marked hypercellularity with closely packed or overlapping cells and loss of the typical vascular pattern; in extreme cases, the myxoid stroma may be absent. Round cells are slightly enlarged but not pleomorphic, with rounded nuclei and scant cytoplasm. Transitional zones with intermediate features between conventional myxoid areas and round-cell foci can also occur [23, 37]. The lesions are stratified by the proportion of round-cell component into low-grade (≤ 5%) and high-grade (> 5%) categories [23], each with distinct biological behaviour and imaging features.

**Fig. 13:**
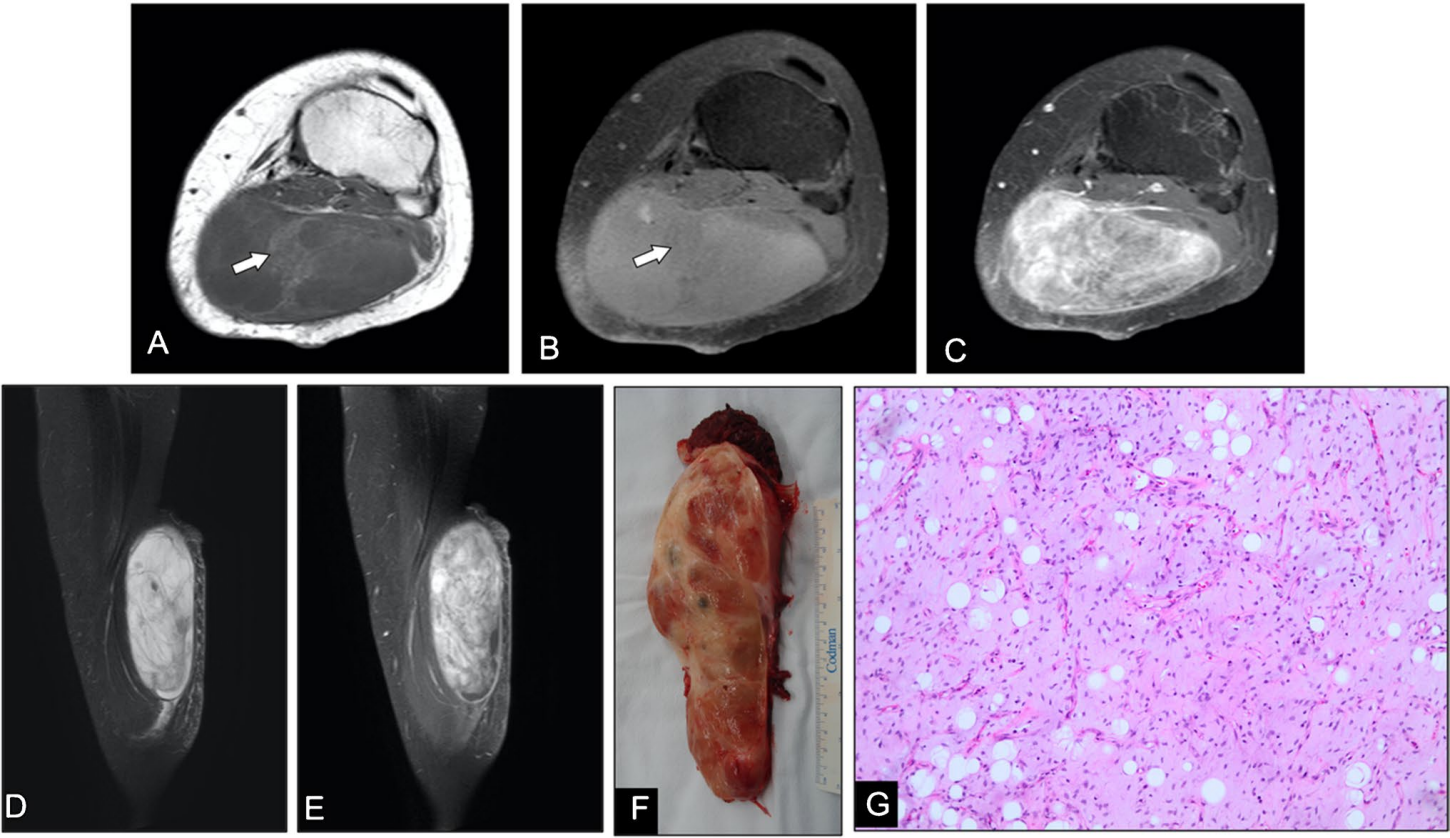
Myxoid liposarcoma of the posterior lower leg in a 39-year-old woman. (**A**) Axial T1-weighted image shows a predominantly low-signal‐intensity mass with focal areas of subtle hyperintensity (arrow), indicating intralesional fat. (**B**) Corresponding fat-suppressed T1-weighted image confirms loss of signal in those hyperintense foci (arrow). Postcontrast axial (**C**) and coronal (**E**) fat-suppressed T1-weighted images demonstrate heterogeneous enhancement throughout the lesion. (**D**) Sagittal fat-suppressed T2-weighted image depicts a markedly hyperintense, “water-like” myxoid matrix. (**F**) Gross specimen reveals a yellow-gray, gelatinous cut surface reflecting high water content. (**G**) Photomicrograph (**H**&E, × 200) shows abundant myxoid stroma with a characteristic plexiform capillary network and scattered lipoblasts. Despite this benign-appearing morphology, prompt and homogeneous contrast enhancement distinguishes myxoid liposarcoma from simple cystic lesions or benign myxoid tumors and underscores the importance of contrast-enhanced imaging in this diagnostic setting

On MRI, low-grade myxoid liposarcoma appears as a well-circumscribed, homogeneous mass with uniformly high signal intensity on fluid-sensitive sequences; its scant fat content may render it indistinguishable from a ganglion cyst on non-enhanced studies. However, prompt and uniform contrast enhancement reliably differentiates it from benign cystic structures (Fig. 13). In contrast, high-grade (round-cell) myxoid liposarcoma exhibits a heterogeneous T2 signal with more conspicuous macroscopic fat than its low-grade counterpart, reflecting its greater cellularity and aggressive potential [56–59].

Metastatic dissemination in myxoid liposarcoma often deviates from the conventional pulmonary-first pattern: extra-pulmonary sites, especially osseous lesions in the spine, may precede lung metastases [52]. These skeletal deposits can be subtle or even occult on CT staging examinations, whereas MRI demonstrates superior sensitivity and specificity relative to CT, PET-CT, and bone scintigraphy for both primary and metastatic disease detection [60–63]. Surgical management remains the mainstay of therapy: wide excision with clear margins is imperative to minimize local recurrence and reduce the risk of distant spread [44].

### Pleomorphic liposarcoma

**Fig. 14:**
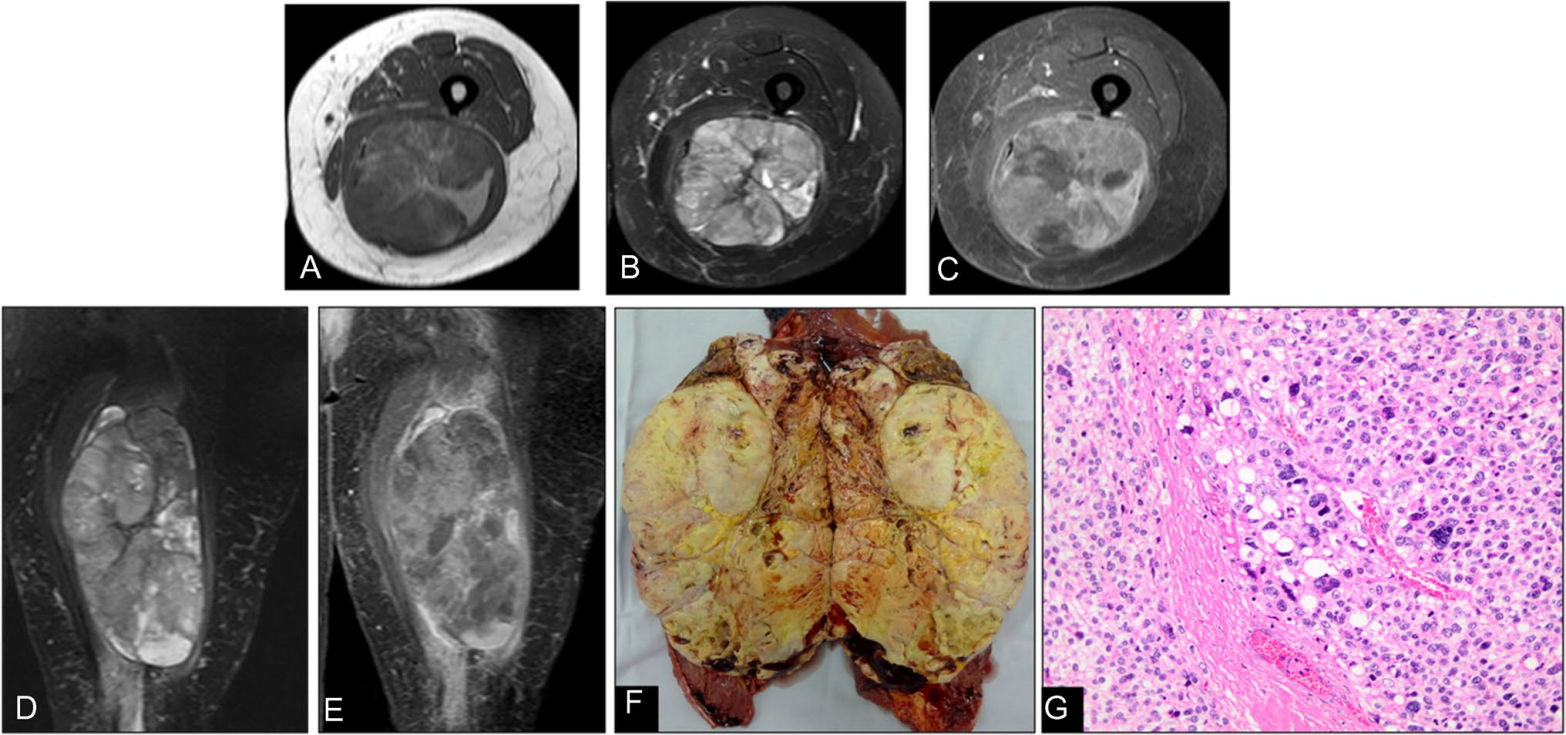
Pleomorphic liposarcoma of the thigh in a 47-year-old woman. (**A**) Axial T1-weighted MR image demonstrates a large heterogeneous soft-tissue mass containing areas of fatty signal. Axial (**B**) and coronal (**D**) fat-suppressed T2-weighted images show mixed high and low signal reflecting necrosis, hemorrhage, and residual fat. Axial (**C**) and coronal (**E**) post-contrast fat-suppressed T1-weighted images reveal marked, inhomogeneous enhancement throughout the lesion. (**F**) Gross specimen photograph displays a variegated cut surface with yellow fatty nodules and firm, pale sarcomatous areas. (**G**) Photomicrograph (**H**&**E**, × 200) shows highly pleomorphic tumor cells with frequent atypical lipoblasts and mitotic figures, confirming pleomorphic liposarcoma

Pleomorphic liposarcoma is an aggressive, high-grade neoplasm associated with the highest rates of local recurrence and distant metastasis among adipocytic tumors. Accounting for only 5%–15% of liposarcomas, it typically affects patients over 50 years old and most commonly involves the lower extremity (56% of cases) [44, 64]. On MRI, pleomorphic liposarcomas demonstrate marked heterogeneity with irregular margins, mixed T1 and T2 signal intensities, and areas of hemorrhage or necrosis (Fig. 14). Adipose tissue comprises less than 25% of the lesion, aiding distinction from more fat-rich subtypes [44, 64]. Contrast-enhanced sequences reveal robust, irregular enhancement within solid components.

Histologically, pleomorphic liposarcoma is a high-grade sarcoma with a mandatory component of lipoblasts, in most cases markedly anaplastic, in variable quantity and distribution. It most often resembles an undifferentiated pleomorphic sarcoma, but some cases are myxofibrosarcoma-like, and approximately 25% show epithelioid morphology, which may mimic, for example, adrenocortical carcinoma [19, 23, 37].

Given their aggressive behavior, pleomorphic liposarcomas require a multimodal treatment approach, typically combining wide surgical excision with adjuvant chemotherapy and radiation therapy to improve local control and reduce metastatic risk [65]. Early recognition of the characteristic imaging and clinical profiles is essential for timely, effective management.

### Myxoid pleomorphic liposarcoma

**Fig. 15:**
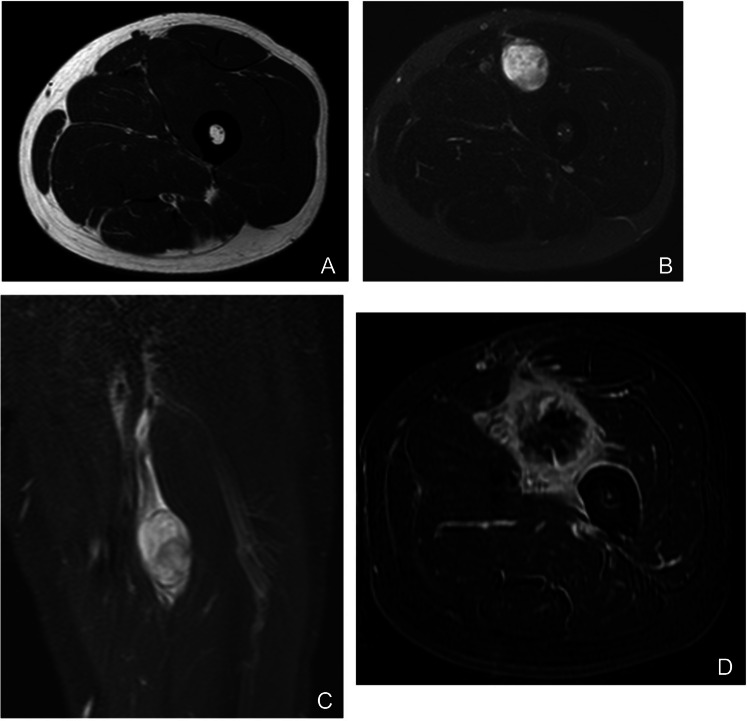
Myxoid pleomorphic liposarcoma of the left thigh. (**A**) Axial T1-weighted image demonstrates a lobulated intramuscular mass in the anterior compartment, predominantly isointense to skeletal muscle with subtle foci of T1 hyperintensity consistent with a fatty component. (**B**) Axial fat-suppressed T2-weighted image shows marked heterogeneous hyperintensity. (**C**) Coronal STIR image delineates the craniocaudal extent of the lesion within the anterior thigh musculature and associated perilesional edema. (**D**) Axial post-contrast T1-weighted subtraction image demonstrates heterogeneous enhancement of the solid nonlipomatous components, with relative lack of enhancement in the more myxoid regions

Myxoid pleomorphic liposarcoma is an exceedingly rare subtype characterized histologically by a combination of myxoid liposarcoma-like and pleomorphic liposarcoma-like components, most commonly affecting young females. The mediastinum is the predominant site, followed by the extremities and head and neck region [19]. No consistent genetic alterations have been identified to date. Clinically, they demonstrate high recurrence rates and early metastasis to lungs, bone, and soft tissues. On MRI, these lesions may mimic conventional myxoid or pleomorphic liposarcomas but often display both myxoid high-T2 signal areas and heterogeneous solid components reflective of pleomorphism (Fig. 15). Management involves aggressive surgical resection with consideration of adjuvant therapies due to their aggressive clinical course [66].

## Discussion

This review explores the spectrum of subtypes of fat-containing soft-tissue lesions, which often share overlapping MRI appearances. The benign nature of lipomas, characterized by their well-circumscribed margins and uniform hyperintense signal on T1-weighted images, often allows for a confident radiologic diagnosis, potentially obviating the need for invasive procedures. Conversely, the imaging findings of liposarcomas are more complex, reflecting their malignant biology and histopathologic heterogeneity. Once identified, appropriate surveillance is critical: the European Society for Medical Oncology (ESMO) recommends that patients with intermediate- to high-grade tumors undergo follow-up every three months during the first two to three years, every six months until year five, and annually thereafter; low-grade tumors should be monitored every four to six months for the first three to five years, then annually [67]. In addition to ESMO recommendations, expert consensus statements emphasize referral of **deep, large, or imaging-atypical adipocytic lesions** to specialized sarcoma centers for multidisciplinary evaluation [18]. Such recommendations reflect the recognized overlap in imaging features between benign lipoma variants and intermediate or malignant adipocytic tumors, particularly in deep-seated locations. While no dedicated American College of Radiology (ACR) guideline specifically addressing lipomatous soft-tissue tumors currently exists, radiologic practice patterns in North America similarly prioritize **multidisciplinary discussion, image-guided biopsy, and histologic confirmation** for lesions that deviate from established benign imaging criteria [17, 68]. This consensus-based approach aims to reduce both underdiagnosis of aggressive tumors and unnecessary surgical intervention for benign entities.

### MRI-based predictive criteria for differentiating atypical lipomatous tumors from benign lipomas

A few studies have established key MRI markers to distinguish benign lipomas from atypical lipomatous tumors (ALTs) and well-differentiated liposarcomas (WDLs) Moran et al. evaluated 79 deep fatty tumors and found that septal thickness ≥ 2 mm, multiple non-fat nodules, and maximum lesion diameter ≥ 12.8 cm yielded 90% sensitivity and 66% specificity for distinguishing atypical lipomatous tumors (ALT)/well-differentiated liposarcoma (WDL) from benign lipomas [8]. Complementing this, Muhib et al.’s systematic review identified consistent MRI predictors—tumor size ≥ 110 mm, patient age > 60 years, lower-extremity location, irregular morphology, incomplete fat suppression, contrast enhancement, septation > 2 mm, and nodularity—reporting sensitivities of 66–100% and specificities of 37–100% [69]. Nagano et al. proposed an MRI-based scoring system, integrating deep location, septal thickness, lesion size, and contrast enhancement. In a cohort of 60 lesions (48 lipomas, 12 ALTs), mean scores were 5.1 for ALT versus 1.7 for lipomas (*P* < 0.001), achieving 100% sensitivity and 77% specificity in guiding surgical margin decisions [9]. Increased intratumoral vascularity in ALTs also correlated with enhancement patterns, highlighting its potential role in assessing malignancy risk [9].

### Functional MRI in lipomatous lesions

The role of contrast enhancement in differentiating benign lipomas from atypical lipomatous tumors remains controversial. Several studies have reported that septal or nodular enhancement may be more frequently observed in ALT/WDLPS; however, enhancement patterns are highly variable and show substantial overlap with benign lipoma variants, particularly involutional or fibrous lipomas. Shannon et al., in a retrospective analysis of 32 peripheral lipomatous tumors, reported no significant improvement in overall diagnostic accuracy (≈53%) with the addition of dynamic contrast-enhanced MRI; however, perfusion metrics such as vascular flow rates and time-to-peak enhancement showed promise for distinguishing benign lipomas from ALT and dedifferentiated liposarcoma [13]. In a prospective single-center study, Gruber et al. [70] demonstrated that the inclusion of contrast-enhanced MRI parameters within a multiparametric scoring system improved lesion classification. Notably, this study emphasized the presence and distribution of enhancement rather than true kinetic analysis, and its findings should be interpreted within the context of a limited sample size. More recently, a large multicenter study by Nalbant et al. [71] found that contrast administration did not significantly improve diagnostic accuracy for distinguishing atypical lipomatous tumors from benign lipomas. These results suggest that routine reliance on contrast enhancement as a discriminator may lead to overestimation of malignancy risk. Taken together, current evidence supports the use of contrast-enhanced MRI as a complementary tool for identifying non-adipose components and guiding targeted biopsy, rather than as a standalone criterion for differentiating benign from intermediate adipocytic tumors.

Diffusion-weighted imaging (DWI) adds limited value in purely lipomatous lesions. According to the 2020 WHO guidelines, apparent diffusion coefficient (ADC) values overlap significantly between lipomas and ALT, and DWI does not substantially enhance lesion characterization or risk stratification in this subgroup [72].

### Application of ST-RADS to lipomatous lesions

The Soft-Tissue Tumor Reporting and Data System (ST-RADS) was developed as a structured MRI-based framework to standardize reporting and risk stratification of soft-tissue masses, including adipocytic tumors. Within this system, benign-appearing lesions (ST-RADS II) typically demonstrate homogeneous fat signal, complete fat suppression, thin septations (< 2 mm), and absence of nodular non-adipose components, whereas higher categories reflect increasing morphologic complexity and risk [10]. While ST-RADS may improve interobserver consistency and facilitate communication with referring clinicians, its current evidence base remains limited. The initial validation studies included relatively small numbers of malignant and indeterminate lipomatous tumors, and therefore the generalizability of the proposed categories should be interpreted with caution [73]. At present, ST-RADS should be regarded as a decision-support and reporting tool, rather than a definitive diagnostic or management guideline.

Importantly, structured reporting systems do not replace expert pattern recognition or multidisciplinary discussion. Lesions categorized as indeterminate or high-risk within ST-RADS still require individualized assessment, correlation with clinical context, and histopathologic confirmation before definitive treatment decisions are made. Broader multicenter validation studies are necessary before widespread adoption of ST-RADS as a standard of care in adipocytic tumor evaluation.

### Recent WHO classification updates and molecular pathology of adipocytic tumors

Adipocytic neoplasms frequently share overlapping MRI characteristics, complicating noninvasive diagnosis [15]. The 2020 WHO classification integrates histologic appearance, anatomic site, and molecular diagnostics to refine adipocytic tumor categorization, emphasizing both tumor behavior and genetic hallmarks [70]. The main changes compared with the 2013 edition were the introduction of myxoid pleomorphic liposarcoma and atypical spindle cell/pleomorphic lipomatous tumor [19, 74].

Potentially confusing terms such as “myxolipoma”, “angiomyxolipoma”, and “dendritic fibromyxolipoma” are not endorsed by the WHO classification. It is preferable to designate benign stromal mucin-rich adipocytic neoplasms with more precise terms, such as “lipoma with myxoid change” or “spindle cell lipoma, myxoid variant”. The new classification also suggests the term “diffuse lipoblastoma” as a synonym for “lipoblastomatosis” [19].

Most liposarcomas can be diagnosed by conventional histology alone, but molecular tests are essential in difficult cases, such as differentiating an ALT/WDLPS with subtle or underrepresented atypia from a lipoma, a DDLPS lacking or with an equivocal well-differentiated component from an undifferentiated sarcoma, or a myxoid liposarcoma from an ALT/WDLPS or lipoma with myxoid change. Modern molecular techniques, particularly FISH and PCR, have largely replaced traditional cytogenetics. ALT/WDLPS and DDLPS are defined by *MDM2* amplification and, cytogenetically, by ring or giant markers chromosomes. Myxoid liposarcoma is characterized by t(12;16)(q13;p11), resulting in the *FUS-DDIT3* fusion; in ~ 3% of cases, the alternative t(12;22)(q13;q22) generates *EWSR1-DDIT3*. Pleomorphic liposarcoma and myxoid pleomorphic liposarcoma, on the other hand, show complex, nonspecific chromosomal aberrations, similar to those of undifferentiated pleomorphic sarcoma [19].

Immunohistochemistry is a valuable adjunct, sometimes serving as a screening tool or an imperfect surrogate. Table 1 summarizes the key molecular alterations across common lipomatous subtypes. Coexpression of MDM2 and CDK4, especially with p16 positivity, provides strong evidence for ALT/WDLPS [75]. For DDLPS, however, greater caution is warranted. Although MDM2 and CDK4 positivity is far more common than in its main histological mimics (undifferentiated pleomorphic sarcoma, malignant peripheral nerve sheath tumor, myxofibrosarcoma) [76], it is recommended to complement a positive immunohistochemical result with molecular analysis, particularly in extremity tumors, where the pre-test probability of DDLPS is considerably lower than in the retroperitoneum [77, 78].

**Table 1:** Imaging, Histologic, and Molecular Features of Fat-Containing Soft-Tissue Tumors, according to 2020 WHO classification

Tumor Subtype	Classification	Key MRI Features	Histologic Features	Molecular Features
Lipoma	Benign	Homogeneous, well-circumscribed fatty mass with complete fat suppression and minimal thin septations	Lobules of mature adipocytes separated by delicate fibrous septa	Characteristic translocations at 12q13-15
Lipoblastoma	Benign	Well-defined, predominantly fat-rich lesion (bright on T1/T2) with heterogeneous post-contrast enhancement	Lobulated clusters of lipoblasts and mature adipocytes within variable myxoid stroma	PLAG1 rearrangements (8q11-13)
Chondroid lipoma	Benign	Predominantly hyperintense on T1 with focal intermediate-signal areas (chondroid matrix); mild, heterogeneous enhancement	Lipoblasts and mature adipocytes embedded in a myxochondroid matrix	C11orf95–MKL2 fusion
Angiolipoma	Benign	Well-marginated subcutaneous fatty mass (bright on T1/T2) with subtle enhancement corresponding to vascular channels	Mature adipocytes interspersed with numerous small blood vessels, sometimes containing fibrin thrombi	No characteristic genetic alterations
Myolipoma	Benign	Large, encapsulated heterogeneous mass; intermixed fat (high T1/T2, suppressed with fat sat) and smooth muscle (intermediate T1, intermediate-to-high T2); occasional coarse calcifications	Admixture of mature adipocytes and smooth muscle cells, often smooth muscle–predominant (2:1); sieve-like pattern; SMA and desmin positive	No characteristic genetic alterations
Hibernoma	Benign	Signal intensity slightly lower than subcutaneous fat on T1-weighted sequences and incomplete suppression on fat-saturated T2-weighted images	Multivacuolated brown adipocytes with abundant mitochondria	Deletions/translocations at 11q13-21; often MEN1 and AIP involvement
Spindle cell/Pleomorphic lipoma	Benign	Well-circumscribed subcutaneous mass (posterior neck/shoulder/back), variable fat content; non-fatty areas isointense to muscle on T1, variable on T2, with delayed enhancement	Mixture of mature adipocytes, bland spindle cells in “school of fish” pattern, rope-like collagen, ± multinucleated floret-like cells; CD34 positive	Recurrent deletions of 13q and/or 16q
Atypical lipomatous tumor/WDL	Intermediate	Fatty mass with thick (> 2 mm), irregular septa and nodular non-fatty foci showing mild enhancement	Mature adipocytes with scattered atypical fusiform or lipogenic cells and occasional lipoblasts	Supernumerary ring/giant chromosomes; amplification of 12q13-15 (MDM2, CDK4, HMGA2)
Dedifferentiated liposarcoma	Malignant	Non-fatty enhancing soft-tissue mass adjacent to fatty ALT/WDL component; irregular margins and heterogeneous signal	Abrupt transition from ALT/WDLPS areas to high-grade spindle or pleomorphic sarcoma	High-level amplifications of 12q13-15 (MDM2, CDK4, HMGA2); CDKN2A deletions
Myxoid liposarcoma	Malignant	Low–intermediate T1 and markedly high T2 signal; internal enhancing septa or nodules within a myxoid background	Abundant myxoid matrix, small fusiform cells, lipoblasts, and chicken-wire capillary network; ± round cells	FUS–DDIT3 fusion (t(12;16)); occasionally EWSR1–DDIT3 (t(12;22))
Pleomorphic liposarcoma	Malignant	Large heterogeneous mass with hemorrhagic/necrotic areas; minimal visible fat; irregular borders	High-grade sarcoma with some highly pleomorphic lipoblasts	TP53 mutations; RB1 deletions
Myxoid pleomorphic liposarcoma	Malignant	Solid heterogeneous lesion combining myxoid and pleomorphic components; variable enhancement	Mixed myxoid liposarcoma-like areas and pleomorphic liposarcoma-like areas	Amplifications on chromosomes 1, 6–8, 18–21; deletions of 13, 16, 17

Crucially, terminology now aligns with surgical considerations: lesions in resectable extremity locations are designated ALT, whereas identical histology in deep sites (e.g., mediastinum, retroperitoneum) is termed WDLPS to reflect increased recurrence risk. This unified framework underscores the seamless integration of imaging findings with molecular assays to guide diagnosis, prognostication, and management.This unified framework underscores the seamless integration of imaging findings with molecular assays to guide diagnosis, prognostication, and management.

### Current trends and future directions

Radiomics and machine-learning applied to MRI and CT have substantially advanced noninvasive classification of lipomatous tumors. Quantitative feature extraction from T1- and T2-weighted images yields radiomic models with AUCs of 0.74–0.89, matching experienced radiologists and reducing unnecessary biopsies [6, 79]. Hybrid workflows combining automated segmentation with expert review improve efficiency and reproducibility [79, 80]. A recent multicenter validation demonstrated high accuracy for a deep-learning segmentation and classification tool across diverse scanners and institutions [80]. Contrast-enhanced CT radiomics predicts MDM2 amplification in retroperitoneal lesions with up to 98% accuracy (AUC) in training and 86% on external validation [68]. Meta-analyses show that combining clinical-imaging scores with radiomic signatures outperforms visual assessment alone, particularly for septal thickness, nodularity, and heterogeneity [81, 82]. Future priorities include standardizing image acquisition, multicenter validation of radiogenomic signatures, and embedding AI decision-support within PACS to personalize surveillance and management.

## Conclusions

MRI remains the cornerstone modality for the noninvasive assessment of fat-containing soft-tissue tumors, particularly for deep-seated, large, or clinically atypical lesions. Careful evaluation of lesion morphology, including size, depth, septal architecture, internal nodularity, and signal heterogeneity, provides the foundation for distinguishing benign lipomas from intermediate and malignant adipocytic tumors.

Despite advances in functional imaging and radiomics, no single imaging feature or technique reliably replaces histopathologic confirmation when atypical features are present. Radiologists should remain vigilant for imaging pitfalls that may lead to false reassurance or overdiagnosis and should prioritize targeted biopsy of non-adipocytic components when indicated.

Familiarity with the 2020 WHO classification and its newly recognized entities is essential for contemporary practice, as imaging findings must increasingly be interpreted in conjunction with molecular and pathologic data. Structured reporting systems and emerging quantitative tools may enhance consistency and communication but should currently be viewed as adjuncts rather than definitive diagnostic standards.

Through an integrated, evidence-based approach that combines conventional MRI assessment with evolving classification frameworks, radiologists can improve diagnostic confidence, optimize patient triage, and contribute meaningfully to multidisciplinary sarcoma care.

## Data Availability

Not applicable.

## References

[1] Myhre-Jensen O. A consecutive 7-year series of 1331 benign soft tissue tumours: clinicopathologic data—comparison with sarcomas. Acta Orthop Scand. 1981;52(3):287–93. 10.3109/17453678109050105. 7282321 10.3109/17453678109050105

[2] Kransdorf MJ, Moser RP Jr, Meis JM, Meyer CA. Fat-containing soft-tissue masses of the extremities. Radiographics. 1991;11(1):81–106. 10.1148/radiographics.11.1.1996399. 1996399 10.1148/radiographics.11.1.1996399

[3] Gupta P, Potti TA, Wuertzer SD, Lenchik L, Pacholke DA. Spectrum of fat-containing soft-tissue masses at MR imaging: the common, the uncommon, the characteristic, and the sometimes confusing. Radiographics. 2016;36(3):753–66. 10.1148/rg.2016150133. 27163592 10.1148/rg.2016150133

[4] Burt AM, Huang BK. Imaging review of lipomatous musculoskeletal lesions. SICOT-J. 2017;3:34. 10.1051/sicotj/2017015. 28474576 10.1051/sicotj/2017015 PMC5418895

[5] Drevelegas A, Pilavaki M, Chourmouzi D. Lipomatous tumors of soft tissue: MR appearance with histological correlation. Eur J Radiol. 2004;50(3):257–67. 10.1016/j.ejrad.2004.01.022. 15145485 10.1016/j.ejrad.2004.01.022

[6] Gitto S, Interlenghi M, Cuocolo R, Salvatore C, Giannetta V, Badalyan J, et al. MRI radiomics-based machine learning for classification of deep-seated lipoma and atypical lipomatous tumor of the extremities. Radiol Med. 2023;128(8):989–98. 10.1007/s11547-023-01657-y. 37335422 10.1007/s11547-023-01657-y PMC10338387

[7] Ballhause TM, Korthaus A, Jahnke M, Frosch KH, Yamamura J, Dust T, et al. Lipomatous tumors: a comparison of MRI-reported diagnosis with histological diagnosis. Diagnostics. 2022;12(5):1281. 10.3390/diagnostics12051281. 35626435 10.3390/diagnostics12051281 PMC9141562

[8] Moran LM, Li CY, Ramirez A, Royuela A. Differentiation of atypical lipomatous tumors from lipomas: our experience with visual analysis of conventional magnetic resonance imaging. J Imaging. 2025;11(2):47. 10.3390/jimaging11020047. 39997549 10.3390/jimaging11020047 PMC11856569

[9] Nagano S, Yokouchi M, Setoguchi T, Ishidou Y, Sasaki H, Shimada H, et al. Differentiation of lipoma and atypical lipomatous tumor by a scoring system: implication of increased vascularity on pathogenesis of liposarcoma. BMC Musculoskelet Disord. 2015;16(1):36. 10.1186/s12891-015-0491-8. 25879189 10.1186/s12891-015-0491-8 PMC4340111

[10] Chhabra A, Ashikyan O, Ratakonda R, Bajaj G, Thakur U, Pezeshk P, et al. Soft-tissue tumor reporting and data system (ST-RADS): MRI reporting guideline with multi-institutional validation study of musculoskeletal extremity tumors. J Tumor Res. 2022;8:179.

[11] Layfield L, Ahlawat S, Morris CD, Levin AS, Fayad LM. Multicenter radiomics study distinguishing lipomas from atypical lipomatous tumors using MRI. Radiology. 2023;307(2):450–8. 10.1148/radiol.230087.

[12] Zhang Q, Liu H, Chen X, Li Z, Zhao L, Wang J, et al. Deep learning–radiomics nomogram for differentiation of well-differentiated liposarcoma and benign lipomas in the retroperitoneum. Eur J Radiol. 2023;159:110739. 10.1016/j.ejrad.2023.110739.

[13] Shannon BA, Ahlawat S, Morris CD, Levin AS, Fayad LM. Do contrast-enhanced and advanced MRI sequences improve diagnostic accuracy for indeterminate lipomatous tumors? Radiol Med. 2022;127(1):90–9. 10.1007/s11547-021-01420-1. 34697728 10.1007/s11547-021-01420-1

[14] Murphey MD, Carroll JF, Flemming DJ, Pope TL, Gannon FH, Kransdorf MJ. From the archives of the AFIP: benign musculoskeletal lipomatous lesions. Radiographics. 2004;24(5):1433–66. 10.1148/rg.245045120. 15371618 10.1148/rg.245045120

[15] Gaskin CM, Helms CA. Lipomas, lipoma variants, and well-differentiated liposarcomas (atypical lipomas): results of MRI evaluations of 126 consecutive fatty masses. AJR Am J Roentgenol. 2004;182(3):733–9. 10.2214/ajr.182.3.1820733. 14975977 10.2214/ajr.182.3.1820733

[16] McTighe S, Chernev I. Intramuscular lipoma: a review of the literature. Orthop Rev (Pavia). 2014;6(4):5618. 10.4081/or.2014.5618. 25568733 10.4081/or.2014.5618 PMC4274454

[17] Crombé A, Kind M, Fadli D, Miceli M, Linck PA, Bianchi G, et al. Soft-tissue sarcoma in adults: imaging appearances, pitfalls and diagnostic algorithms. Diagn Interv Imaging. 2023;104(5):207–20. 10.1016/j.diii.2023.02.005. 36567193 10.1016/j.diii.2022.12.001

[18] Moulin B, Messiou C, Crombe A, Kind M, Hohenberger P, Rutkowski P, et al. Diagnosis strategy of adipocytic soft-tissue tumors in adults: a consensus from European experts. Eur J Surg Oncol. 2022;48(3):518–25. 10.1016/j.ejso.2021.10.009. 34688512 10.1016/j.ejso.2021.10.009

[19] WHO Classification of Tumours Editorial Board. Soft tissue and bone tumours, vol. 3. 5th ed. Lyon: International Agency for Research on Cancer; 2020.

[20] Chung EB, Enzinger FM. Benign lipoblastomatosis: an analysis of 35 cases. Cancer. 1973;32(2):482–92. 10.1002/1097-0142(197308)32:2<482::AID-CNCR2820320229>3.0.CO;2-E. 4353020 10.1002/1097-0142(197308)32:2<482::aid-cncr2820320229>3.0.co;2-e

[21] Dilley AV, Patel DL, Hicks MJ, Brandt ML. Lipoblastoma: pathophysiology and surgical management. J Pediatr Surg. 2001;36(2):229–31. 10.1053/jpsu.2001.20060. 11150471 10.1053/jpsu.2001.20060

[22] Chun YS, Kim WK, Park KW, Lee SC, Jung SE. Lipoblastoma. J Pediatr Surg. 2001;36(6):905–7. 10.1053/jpsu.2001.23969. 11381423 10.1053/jpsu.2001.23969

[23] Lindberg MR. Diagnostic pathology: soft tissue tumors. 2nd ed. Philadelphia: Elsevier; 2015.

[24] Degnan AJ, Jelinek JS, Murphey MD. Lipoblastoma: computed tomographic and magnetic resonance imaging features correlate with tumor behavior and pathology. Pediatr Radiol. 2021;51(4):614–21. 10.1007/s00247-020-04882-z. 33151344 10.1007/s00247-020-04882-z

[25] Sheybani EF, Eutsler EP, Navarro OM. Fat-containing soft-tissue masses in children. Pediatr Radiol. 2016;46(13):1760–73. 10.1007/s00247-016-3690-z. 27866258 10.1007/s00247-016-3690-z

[26] Thway K, Flora RS, Fisher C. Chondroid lipoma: an update and review. Ann Diagn Pathol. 2012;16(3):230–4. 10.1016/j.anndiagpath.2012.01.002. 22607659 10.1016/j.anndiagpath.2012.01.002

[27] Boets A, Van Mieghem IM, Sciot R, Van Breuseghem I. Chondroid lipoma of the trunk: MRI appearance and pathologic correlation. Skeletal Radiol. 2004;33(11):666–9. 10.1007/s00256-004-0774-x. 15133639 10.1007/s00256-004-0774-x

[28] Hyzy MD, Hogendoorn PC, Bloem JL, De Schepper AM. Chondroid lipoma: findings on radiography and MRI. Eur Radiol. 2006;16(10):2373–6. 10.1007/s00330-006-0243-0. 16924441 10.1007/s00330-006-0243-0

[29] Green RA, Cannon SR, Flanagan AM. Chondroid lipoma: correlation of imaging findings and histopathology of an unusual benign lesion. Skeletal Radiol. 2004;33(11):670–3. 10.1007/s00256-004-0818-2. 15351916 10.1007/s00256-004-0818-2

[30] Kransdorf MJ, Larsen BT, Goulding KA, Cumsky JL, Hwang S, Long JR. Angiolipoma: a review of 778 lesions in 344 patients. Skeletal Radiol. 2023;52(3):541–52. 10.1007/s00256-022-04075-9. 35668116 10.1007/s00256-022-04075-9

[31] Bang M, Kang BS, Hwang JC, Weon YC, Choi SH, Shin SH, et al. Ultrasonographic analysis of subcutaneous angiolipoma. Skeletal Radiol. 2012;41(9):1055–9. 10.1007/s00256-011-1309-x. 22064985 10.1007/s00256-011-1309-x

[32] Shin YS, Kim YJ, Park IS, Chu YC, Kim JH, Lee HY, et al. Sonographic differentiation between angiolipomas and superficial lipomas. J Ultrasound Med. 2016;35(11):2421–9. 10.7863/ultra.15.08050. 27738296 10.7863/ultra.15.08050

[33] Furlong MA, Fanburg-Smith JC, Miettinen M. The morphologic spectrum of hibernoma: a clinicopathologic study of 170 cases. Am J Surg Pathol. 2001;25(6):809–14. 10.1097/00000478-200106000-00014. 11395560 10.1097/00000478-200106000-00014

[34] Lee JC, Gupta A, Saifuddin A, Flanagan A, Skinner JA, Briggs TW, et al. Hibernoma: MRI features in eight consecutive cases. Clin Radiol. 2006;61(12):1029–34. 10.1016/j.crad.2006.05.018. 17097424 10.1016/j.crad.2006.05.018

[35] Liu W, Bui MM, Cheong D, Caracciolo JT. Hibernoma: comparing imaging appearance with more commonly encountered benign or low-grade lipomatous neoplasms. Skeletal Radiol. 2013;42(8):1073–8. 10.1007/s00256-013-1583-x. 23385517 10.1007/s00256-013-1583-x

[36] Beals C, Rogers A, Wakely P, Mayerson JL, Scharschmidt TJ. Hibernomas: a single-institution experience and review of literature. Med Oncol. 2014;31(1):769. 10.1007/s12032-013-0769-3. 24248814 10.1007/s12032-013-0769-3

[37] Goldblum JR, Folpe AL, Weiss SW. Enzinger and Weiss’s Soft Tissue Tumors. 6th ed. Philadelphia: Elsevier; 2013.

[38] Kamaci S, Doral MN, Ergen FB, Yucekul A, Cil A. Lipoma arborescens of the knee. Knee Surg Sports Traumatol Arthrosc. 2015;23(8):2196–201. 10.1007/s00167-014-2996-3. 24752536 10.1007/s00167-014-2996-3

[39] Howe BM, Wenger DE. Lipoma arborescens: comparison of typical and atypical disease presentations. Clin Radiol. 2013;68(12):1220–6. 10.1016/j.crad.2013.07.002. 23969149 10.1016/j.crad.2013.07.002

[40] Ryu KN, Jaovisidha S, Schweitzer M, Motta AO, Resnick D. MR imaging of lipoma arborescens of the knee joint. AJR Am J Roentgenol. 1996;167(5):1229–32. 10.2214/ajr.167.5.8911186. 8911186 10.2214/ajr.167.5.8911186

[41] Tahiri Y, Xu L, Kanevsky J, Luc M. Lipofibromatous hamartoma of the median nerve: a comprehensive review and systematic approach to evaluation, diagnosis, and treatment. J Hand Surg Am. 2013;38(10):2055–67. 10.1016/j.jhsa.2013.03.022. 23684521 10.1016/j.jhsa.2013.03.022

[42] Mohanty CB, Midha R. Lipomatosis of nerve. World Neurosurg. 2014;82(3–4):331–2. 10.1016/j.wneu.2013.10.001. 24103548 10.1016/j.wneu.2013.10.001

[43] Coelho RDS, Simão MN, Trad CS. Fibrolipomatous hamartoma and macrodystrophia lipomatosa: imaging and clinical data analysis of four cases and review of the literature. Radiol Bras. 2002;35(5):287–91.

[44] Murphey MD, Arcara LK, Fanburg-Smith J. From the archives of the AFIP: imaging of musculoskeletal liposarcoma with radiologic-pathologic correlation. Radiographics. 2005;25(5):1371–95. 10.1148/rg.255055106. 16160117 10.1148/rg.255055106

[45] Kransdorf MJ, Bancroft LW, Peterson JJ, Murphey MD, Foster WC, Temple HT. Imaging of fatty tumors: distinction of lipoma and well-differentiated liposarcoma. Radiology. 2002;224(1):99–104. 10.1148/radiol.2241011536. 12091667 10.1148/radiol.2241011113

[46] Henze J, Bauer S. Liposarcomas. Hematol Oncol Clin North Am. 2013;27(5):939–55. 10.1016/j.hoc.2013.07.010. 24093169 10.1016/j.hoc.2013.07.010

[47] Crago AM, Singer S. Clinical and molecular approaches to well differentiated and dedifferentiated liposarcoma. Curr Opin Oncol. 2011;23(4):373–8. 10.1097/CCO.0b013e32834796e6. 21552124 10.1097/CCO.0b013e32834796e6 PMC3253354

[48] Weiss SW, Rao VK. Well-differentiated liposarcoma (atypical lipoma) of deep soft tissue of the extremities, retroperitoneum, and miscellaneous sites: a follow-up study of 92 cases with analysis of the incidence of dedifferentiation. Am J Surg Pathol. 1992;16(11):1051–8. 10.1097/00000478-199211000-00003. 1471725 10.1097/00000478-199211000-00003

[49] Jour G, et al. Low-grade dedifferentiated liposarcoma: a clinicopathologic analysis of 19 cases. Mod Pathol. 2015;28:1230–8. 10.1038/modpathol.2015.80.

[50] Graham RP, et al. Low-grade dedifferentiated liposarcoma: a detailed clinicopathologic and outcome study of 31 cases. Mod Pathol. 2023;36:100039. 10.1016/j.modpat.2023.100039.36853789

[51] Le Guellec S, Chibon F, Ouali M, Perot G, Decouvelaere AV, Robin YM, et al. Are peripheral purely undifferentiated pleomorphic sarcomas with MDM2 amplification dedifferentiated liposarcomas? Am J Surg Pathol. 2014;38(3):293–304. 10.1097/PAS.0000000000000131. 24525499 10.1097/PAS.0000000000000131

[52] Hong SH, Kim KA, Woo OH, Jeong SY, Cho SH, Lee SH, et al. Dedifferentiated liposarcoma of retroperitoneum: spectrum of imaging findings in 15 patients. Clin Imaging. 2010;34(3):203–10. 10.1016/j.clinimag.2009.12.025. 20416485 10.1016/j.clinimag.2009.12.025

[53] Kawaguchi M, Kato H, Kobayashi K, Miyazaki T, Nagano A, Noda Y, et al. MRI findings to differentiate musculoskeletal dedifferentiated liposarcoma from atypical lipomatous tumor. Radiol Med. 2022;127(12):1383–9. 10.1007/s11547-022-01547-9. 36350422 10.1007/s11547-022-01547-9

[54] Löwenthal D, Zeile M, Niederhagen M, Stenzhorn H, Bannas P, Beer AJ, et al. Differentiation of myxoid liposarcoma by magnetic resonance imaging: a histopathologic correlation. Acta Radiol. 2014;55(8):952–60. 10.1177/0284185113508114. (**PMID:24123962**). 24123962 10.1177/0284185113508114

[55] Lemeur M, Mattei JC, Souteyrand P, Chagnaud C, Curvale G, Rochwerger A. Prognostic factors for the recurrence of myxoid liposarcoma: 20 cases with up to 8 years follow-up. Orthop Traumatol Surg Res. 2015;101(1):103–7. 10.1016/j.otsr.2014.09.024. 25583234 10.1016/j.otsr.2014.09.024

[56] Scalas G, Parmeggiani A, Martella C, Tuzzato G, Bianchi G, Facchini G, et al. Magnetic resonance imaging of soft tissue sarcoma: features related to prognosis. Eur J Orthop Surg Traumatol. 2021;31(8):1567–75. 10.1007/s00590-021-03003-2. 34052920 10.1007/s00590-021-03003-2

[57] Barile A, Zugaro L, Catalucci A, Caulo M, Di Cesare E, Splendiani A, et al. Soft tissue liposarcoma: histological subtypes. MRI and CT findings Radiol Med. 2002;104(3):140–9. 12471362

[58] Durr HR, Rauh J, Baur-Melnyk A, Knösel T, Lindner L, Roeder F, et al. Myxoid liposarcoma: local relapse and metastatic pattern in 43 patients. BMC Cancer. 2018;18(1):304. 10.1186/s12885-018-4226-8. 29558901 10.1186/s12885-018-4226-8 PMC5859402

[59] Gorelik N, Reddy SMV, Turcotte RE, Isler MH, Clarkson PW, Ferguson PC, et al. Early detection of metastases using whole-body MRI for initial staging and routine follow-up of myxoid liposarcoma. Skeletal Radiol. 2018;47(3):369–79. 10.1007/s00256-017-2845-9. 29275455 10.1007/s00256-017-2845-9

[60] Gouin F, Renault A, Bertrand-Vasseur A, Biau D, Mascard E, Anract P, et al. Early detection of multiple bone and extra-skeletal metastases by body magnetic resonance imaging (BMRI) after treatment of myxoid/round-cell liposarcoma (MRCLS). Eur J Surg Oncol. 2019;45(12):2431–6. 10.1016/j.ejso.2019.08.014. 31447287 10.1016/j.ejso.2019.08.014

[61] Noble JL, Moskovic E, Fisher C, Judson I. Imaging of skeletal metastases in myxoid liposarcoma. Sarcoma. 2010;2010:262361. 10.1155/2010/262361. 20369068 10.1155/2010/262361 PMC2847760

[62] Stevenson JD, Watson JJ, Cool P, Leahy M, Laitinen M, Parry M, et al. Whole-body magnetic resonance imaging in myxoid liposarcoma: a useful adjunct for the detection of extra-pulmonary metastatic disease. Eur J Surg Oncol. 2016;42(4):574–80. 10.1016/j.ejso.2015.12.011. 26831007 10.1016/j.ejso.2015.12.011

[63] El Ouni F, Jemni H, Trabelsi A, Ben Maitigue M, Bouaziz MC, Ladeb MF, et al. Liposarcoma of the extremities: MR imaging features and their correlation with pathologic data. Orthop Traumatol Surg Res. 2010;96(8):876–83. 10.1016/j.otsr.2010.05.010. 20934400 10.1016/j.otsr.2010.05.010

[64] Oliveira AM, Nascimento AG. Pleomorphic liposarcoma. Semin Diagn Pathol. 2001;18(4):274–85. 11757868

[65] Dei Tos AP. Liposarcomas: diagnostic pitfalls and new insights. Histopathology. 2014;64(1):38–52. 10.1111/his.12311. 24118009 10.1111/his.12311

[66] Matsumoto K, Takada M, Okabe H, Ishizawa M. Foci of signal intensities different from fat in well-differentiated liposarcoma and lipoma: correlation between MR and histological findings. Clin Imaging. 2000;24(1):38–43. 10.1016/s0899-7071(00)00160-1. 11120416 10.1016/s0899-7071(00)00160-1

[67] Casali PG, Abecassis N, Aro HT, Bauer S, Biagini R, Bielack S, et al. Soft tissue and visceral sarcomas: ESMO–EURACAN–GENTURIS clinical practice guidelines for diagnosis, treatment and follow-up. Ann Oncol. 2021;32(11):1348–65. 10.1016/j.annonc.2021.07.006. 34303806 10.1016/j.annonc.2021.07.006

[68] Spaanderman DJ, Hakkesteegt SN, Hanff DF, Schut ARW, Schiphouwer LM, Vos M, et al. Multi-center external validation of an automated method segmenting and differentiating atypical lipomatous tumors from lipomas using radiomics and deep-learning on MRI. eClin Med. 2024;76:102802. 10.1016/j.eclinm.2024.102802. 10.1016/j.eclinm.2024.102802 PMC11440245 39351025

[69] Muhib M, Abidi SLF, Ahmed U, Afzal A, Farooqui A, Jamil OBK, et al. Use of radiologic imaging to differentiate lipoma from atypical lipomatous tumor/well-differentiated liposarcoma: systematic review. SAGE Open Med. 2024;12:20503121241293496. 10.1177/20503121241293496. 39526094 10.1177/20503121241293496 PMC11549689

[70] Gruber L, Kremser C, Zelger B, Schwabegger A, Josip E, Dammerer D, et al. Evaluation of classic and quantitative imaging features in the differentiation of benign and atypical lipomatous soft tissue tumors using a standardized multiparametric MRI protocol: a prospective single-centre study in 45 patients. Curr Oncol. 2023;30(3):3315–28. 10.3390/curroncol30030252. 36975465 10.3390/curroncol30030252 PMC10047222

[71] Nalbant H, Abdelhafez YG, Bateni C, Godinez F, Lee S, Zhang M, et al. MRI of atypical lipomatous tumor: does contrast help? A multicenter study. Skeletal Radiol. 2025;54(12):2681–93. 10.1007/s00256-025-04957-8. 40471269 10.1007/s00256-025-04957-8 PMC12552331

[72] Ahlawat S, Fayad LM. Updates to the WHO classification of soft tissue tumors: implications for imaging specialists. Pol J Radiol. 2020;85:e417–23. 10.5114/pjr.2020.94093.

[73] Wilson MP, Haidey J, Murad MH, Sept L, Low G. Diagnostic accuracy of CT and MR features for detecting atypical lipomatous tumors and malignant liposarcomas: a systematic review and meta-analysis. Eur Radiol. 2023;33(12):8605–16. 10.1007/s00330-023-09916-2. 37439933 10.1007/s00330-023-09916-2

[74] Fletcher CDM, Bridge JA, Hogendoorn PCW, Mertens F, editors. *WHO* Classification of Tumours of Soft Tissue and Bone. 4th ed. Lyon: IARC Press; 2013.

[75] Thway K, Flora R, Shah C, Olmos D, Fisher C. Diagnostic utility of p16, CDK4, and MDM2 as an immunohistochemical panel in distinguishing well-differentiated and dedifferentiated liposarcomas from other adipocytic tumors. Am J Surg Pathol. 2012;36(3):462–9. 10.1097/PAS.0b013e3182417330. 22301498 10.1097/PAS.0b013e3182417330

[76] Binh MB, Sastre-Garau X, Guillou L, de Pinieux G, Terrier P, Lagacé R, et al. MDM2 and CDK4 immunostainings are useful adjuncts in diagnosing well-differentiated and dedifferentiated liposarcoma subtypes: a comparative analysis of 559 soft tissue neoplasms with genetic data. Am J Surg Pathol. 2005;29(10):1340–7. 10.1097/01.pas.0000170343.09562.39. 16160477 10.1097/01.pas.0000170343.09562.39

[77] Le Guellec S, Chibon F, Ouali M, Perot G, Decouvelaere AV, Robin YM, et al. Are peripheral purely undifferentiated pleomorphic sarcomas with MDM2 amplification dedifferentiated liposarcomas? Am J Surg Pathol. 2014;38(3):293–304. 10.1097/PAS.0000000000000131. 24525499 10.1097/PAS.0000000000000131

[78] Sirvent N, Coindre JM, Maire G, Hostein I, Keslair F, Guillou L, et al. Detection of MDM2-CDK4 amplification by fluorescence in situ hybridization in 200 paraffin-embedded tumor samples: utility in diagnosing adipocytic lesions and comparison with immunohistochemistry and real-time PCR. Am J Surg Pathol. 2007;31(10):1476–89. 10.1097/PAS.0b013e3180581fff. 17895748 10.1097/PAS.0b013e3180581fff

[79] Vos M, Starmans MPA, Timbergen MJM, van der Voort SR, Padmos GA, Kessels W, et al. Radiomics approach to distinguish between well-differentiated liposarcomas and lipomas on MRI. Br J Surg. 2019;106(13):1800–9. 10.1002/bjs.11410. 31747074 10.1002/bjs.11410 PMC6899528

[80] Haidey J, Low G, Wilson MP. Radiomics-based approaches outperform visual analysis for differentiating lipoma from atypical lipomatous tumors: a review. Skeletal Radiol. 2023;52(6):1089–100. 10.1007/s00256-022-04232-0. 36385583 10.1007/s00256-022-04232-0

[81] Xu J, Miao L, Wang CX, Wang HH, Wang QZ, Li M, et al. Preoperative contrast-enhanced CT-based deep learning radiomics model for distinguishing retroperitoneal lipomas and well-differentiated liposarcomas. Acad Radiol. 2024;31(12):5042–53. 10.1016/j.acra.2024.06.035. 39003228 10.1016/j.acra.2024.06.035

[82] Nardo L, Abdelhafez YG, Acquafredda F, Schirò S, Wong AL, Sarohia D, et al. Qualitative evaluation of MRI features of lipoma and atypical lipomatous tumor: results from a multicenter study. Skeletal Radiol. 2020;49(6):1005–14. 10.1007/s00256-020-03372-5. 31965239 10.1007/s00256-020-03372-5

